# Creating Cycling-Friendly Environments for Children: Which Micro-Scale Factors Are Most Important? An Experimental Study Using Manipulated Photographs

**DOI:** 10.1371/journal.pone.0143302

**Published:** 2015-12-01

**Authors:** Ariane Ghekiere, Benedicte Deforche, Lieze Mertens, Ilse De Bourdeaudhuij, Peter Clarys, Bas de Geus, Greet Cardon, Jack Nasar, Jo Salmon, Jelle Van Cauwenberg

**Affiliations:** 1 Department of Public Health, Faculty of Medicine and Health Sciences, Ghent University, B-9000, Ghent, Belgium; 2 Department of Human Biometry and Biomechanics, Faculty of Physical Education and Physical Therapy, Vrije Universiteit Brussel, B-1050, Brussels, Belgium; 3 Fund for Scientific Research Flanders (FWO), B-1000, Brussels, Belgium; 4 Department of Movement and Sport Sciences, Faculty of Medicine and Health Sciences, Ghent University, B-9000, Ghent, Belgium; 5 Department of Human Physiology, Faculty of Physical Education and Physical Therapy, Vrije Universiteit Brussel, B-1050, Brussels, Belgium; 6 Ohio State University, City and Regional Planning, Columbus, OH, 43210, United States of America; 7 Centre for Physical Activity and Nutrition Research, School of Exercise and Nutrition Science, Deakin University, Melbourne, Australia; University of New South Wales, AUSTRALIA

## Abstract

**Background:**

Increasing participation in transportation cycling represents a useful strategy for increasing children’s physical activity levels. Knowledge on how to design environments to encourage adoption and maintenance of transportation cycling is limited and relies mainly on observational studies. The current study experimentally investigates the relative importance of micro-scale environmental factors for children’s transportation cycling, as these micro-scale factors are easier to change within an existing neighborhood compared to macro-scale environmental factors (i.e. connectivity, land-use mix, …).

**Methods:**

Researchers recruited children and their parents (n = 1232) via 45 randomly selected schools across Flanders and completed an online questionnaire which consisted of 1) demographic questions; and 2) a choice-based conjoint (CBC) task. During this task, participants chose between two photographs which we had experimentally manipulated in seven micro-scale environmental factors: type of cycle path; evenness of cycle path; traffic speed; traffic density; presence of speed bumps; environmental maintenance; and vegetation. Participants indicated which route they preferred to (let their child) cycle along. To find the relative importance of these micro-scale environmental factors, we conducted Hierarchical Bayes analyses.

**Results:**

Type of cycle path emerged as the most important factor by far among both children and their parents, followed by traffic density and maintenance, and evenness of the cycle path among children. Among parents, speed limits and maintenance emerged as second most important, followed by evenness of the cycle path, and traffic density.

**Conclusion:**

Findings indicate that improvements in micro-scale environmental factors might be effective for increasing children’s transportation cycling, since they increase the perceived supportiveness of the physical environment for transportation cycling. Investments in creating a clearly designated space for the young cyclist, separated from motorized traffic, appears to be the most effective way to increase perceived supportiveness. Future research should confirm our laboratory findings with experimental on-site research.

## Introduction

Most children in Europe do not achieve the recommended hour of moderate to vigorous physical activity per day to obtain health benefits [1, 2]. However, children’s physical activity levels track to levels at older ages, indicating the need to promote physical activity during childhood [3]. Since 2005, active transport (i.e. walking or cycling to a destination within the neighborhood) has been identified as an important source of physical activity among children [2, 4]. In some European countries such as Belgium, Germany, Denmark and The Netherlands, people more often cycle than walk for transport [5–7]. Children can cover larger distances cycling compared to walking, making cycling an attractive, inexpensive and accessible transportation mode. Children who regularly cycle for transport have better cardiovascular health [8, 9], have better physical fitness [10], have a lower body mass index [11, 12] and a healthier body composition [13] compared to those driven to their destinations. In addition to its health benefits, cycling can reduce traffic congestion and associated traffic-related air pollution [14]. Despite the many benefits associated with cycling for transport [15], it remains a challenge to increase cycling for transport rates among Flemish primary school aged children [16]. Currently, 40% of Flemish 10–12 year old children living within 3 km from school never cycle to school [17].

From a socio-ecological approach, that emphasizes the importance of individual (e.g. attitude, social support) and environmental factors (e.g. quality of the cycling infrastructure, traffic speed) [18], research should identify physical environmental factors that may support cycling. Handy and colleagues (2014) emphasized the need for studies identifying key factors influencing transportation cycling. Such studies can inform planners on how to conduct the most effective and efficient structural changes to the physical environment [19]. Identifying these key factors by extensive exploratory research can help to avoid wasting limited resources on ineffective changes to the physical environment. Consider three kinds of environmental factors, (1) fixed factors (like street layout including type of buildings) which are more permanent and thus difficult and expensive to change, (2) semi-fixed factors which are more movable objects placed in the environment (like street amenities including traffic regulations and cycling facilities), and (3) movable features, which refers to humans and objects (such as a chair) not attached to the environment. The present paper focuses on micro-scale semi-fixed environmental factors, such as the evenness of cycle path, the amount of vegetation and the speed restrictions, which are street characteristics that are the responsibility of local actors within the community.

Cross-sectional studies have examined the associations between physical environmental characteristics and children’s transportation cycling [20–22]. For example, studies consistently find that as the distance to destinations increases the likelihood of transportation cycling decreases [20]. Additionally, studies have found that children’s cycling increases with the perceived traffic safety [23]. Perceived traffic safety can be influenced by a broad range of micro-scale environmental factors, such as the type of cycling infrastructure (cycle path, cycle lane), the degree of separation between motorized traffic and the cycle path, traffic speed, traffic density, and the evenness of cycling infrastructure [24]. Parental perceptions of traffic safety may even be more important as this has been considered as an important factor determining children’s independent mobility [25]. Findings from studies examining associations between micro-scale environmental characteristics and children’s transportation cycling are inconsistent, but this may result from measurement issues [22].

Previous studies have mainly used self-report questionnaires, asking the participant to describe characteristics within their neighborhood. This method has several limitations. First, they require the participant to recall specific, detailed environmental factors while not in that environment which may result in a recall bias [26, 27]. Additionally, what people say and recall may well differ from what they noticed when experiencing the environment, and how it affects their cycling behavior. Second, there is no consensus about the best definition of the “neighborhood” when asking participants to describe physical elements within their neighbourhood [28, 29]. Finally, physical characteristics of a neighbourhood may co-vary, i.e. some environmental factors tend to occur together. For example, benches are more prevalent in neighborhoods with a lot of vegetation. This co-variation of environmental factors has been identified as a topic which needs further investigation [20].

Photographs can overcome these limitations in that they neither require participants to recall an environment nor respond based on their own definition of their neighbourhood. Photographs also allow controlled manipulations of physical environmental factors (e.g. evenness of cycle path, speed limits etc.) to test the potential causal relationships between each environmental factor and participants’ preferences. The feasibility of using photographs has been tested in some pilot studies [30–34].

While natural experiments are the best way to determine causal effects of structural changes on cycling behavior, structural changes in the environment are very expensive and time consuming. However, extensive exploratory research is needed in order to inform urban planners or other researchers on which structural changes will be most likely to increase children’s transportation cycling. Photographs allow one to simulate and test these changes relatively quickly and at low cost. Therefore, the current study uses manipulated photographs in a laboratory setting, to examine which environmental factors have the most influence on the perceived supportiveness of a street for children’s transportation cycling. In this study, both children and their parents participated, as both may influence the child’s actual transport mode choice.

We gave special attention to identify whether the effect of micro-scale environmental factors is equal across different subgroups [35]. Perhaps, the existence of subgroups might explain the inconsistent associations between the micro-scale environmental factors and children’s cycling for transport [22]. Insight into how individual factors may influence the importance of specific environmental factors is lacking. However, this knowledge is essential for tailoring future changes in the physical environment to the needs of different individuals. Subgroups differing in preferences for a particular environmental factor may exist based on the amount of transportation cycling of the individual and age of the child. When children grow older, the importance of safety may decrease due to their greater autonomy [20]. Additional differences may be observed according to sex [36], as girls/mothers may be more concerned about traffic safety, while for boys/fathers this may be less important. Other differences may be observed according to children’s perceived cycling skills, as children who feel less confident about cycling, may focus on the evenness of the cycle path, while for more experienced/more confident cyclists other environmental factors might be more important. Finally, it is hypothesized that specific micro-scale environmental factors might be of importance depending on the neighborhood in which they live. Therefore, the current study firstly investigates which micro-scale environmental factors are most important to create cycling-friendly streets for the total sample of both children and parents, and secondly, identifies whether subgroups exist which are characterized by a preference for specific micro-scale environmental factors.

## Materials and Methods

### Recruitment and procedures

Researchers recruited children via randomly selected primary schools across Flanders. The researcher visited each school twice. During the first visit, children from the 5^th^ and 6^th^ grade (primary school, 10–12 yrs old) received a letter including information of the study, a link to the website of the study and a personalized login which enabled parents to participate in the study, by completing an online questionnaire at home. Parents had to give active written consent for their child to participate at school. After one week, the researchers returned to the schools to collect the informed consents and the children completed an online questionnaire at school. School visits were conducted in November and December 2014, while the parental questionnaire closed at the end of January 2015.

Prior to data collection, sample size calculations determined that the study needed a sample of 1000 children and 1000 parents to meet its study aims [37]. A pilot study [34] suggested that about half of the parents of the children who complete the survey in school would complete the parental survey at home. Therefore, we needed to recruit 2000 children to get sufficient parental participants. Therefore, researchers telephoned one-hundred and nine primary schools randomly chosen across Flanders of which forty-five agreed to participate in the study (participation rate = 41%). The Ethics Committee of the University Hospital of Ghent University approved the study protocol.

### Development of the photographs

Prior to data collection, a set of 1945 panoramic photographs was manipulated with Adobe Photoshop software®. These photographs depicted a typical urban street in Flanders where children could cycle. This street was manipulated on the following seven micro-scale environmental factors: type of cycle path; evenness of the cycle path; traffic speed; amount of vegetation; maintenance; traffic density; and presence of a speed bump. Based on previous research, these micro-environmental factors were hypothesized to have an influence on the supportiveness of a street for children for cycling [24, 38, 39]. As summarized in Table 1, each micro-environmental factor could be depicted at different levels, ranging from an unsupportive level to a more supportive level.

**Table 1:** Overview of the included environmental factors and specific levels in the photographs.

Type of cycle path	No cycle path
	Cycle path separated from traffic with lines, not separated from walking path (advisory cycle path)
	Cycle path separated from traffic with a curb, not separated from walking path
	Cycle path separated from traffic with a hedge, not separated from walking path
	Cycle path separated from traffic with a curb, separated from walking path by colour
	Cycle path separated from traffic with a hedge, separated from walking path by colour
**Evenness of cycle path**	Very uneven
	Moderately uneven
	Even
**Traffic speed**	50 km/h
	30 km/h
**Vegetation**	No trees
	Two trees
	Four trees
**Maintenance**	Bad upkeep (much graffiti and litter)
	Moderate upkeep (a bit of graffiti and litter)
	Good upkeep (no graffiti or litter)
**Traffic density**	4 cars + truck
	3 cars
	1 car
**Speed bump**	Absent
	Present

**Fig 1:**
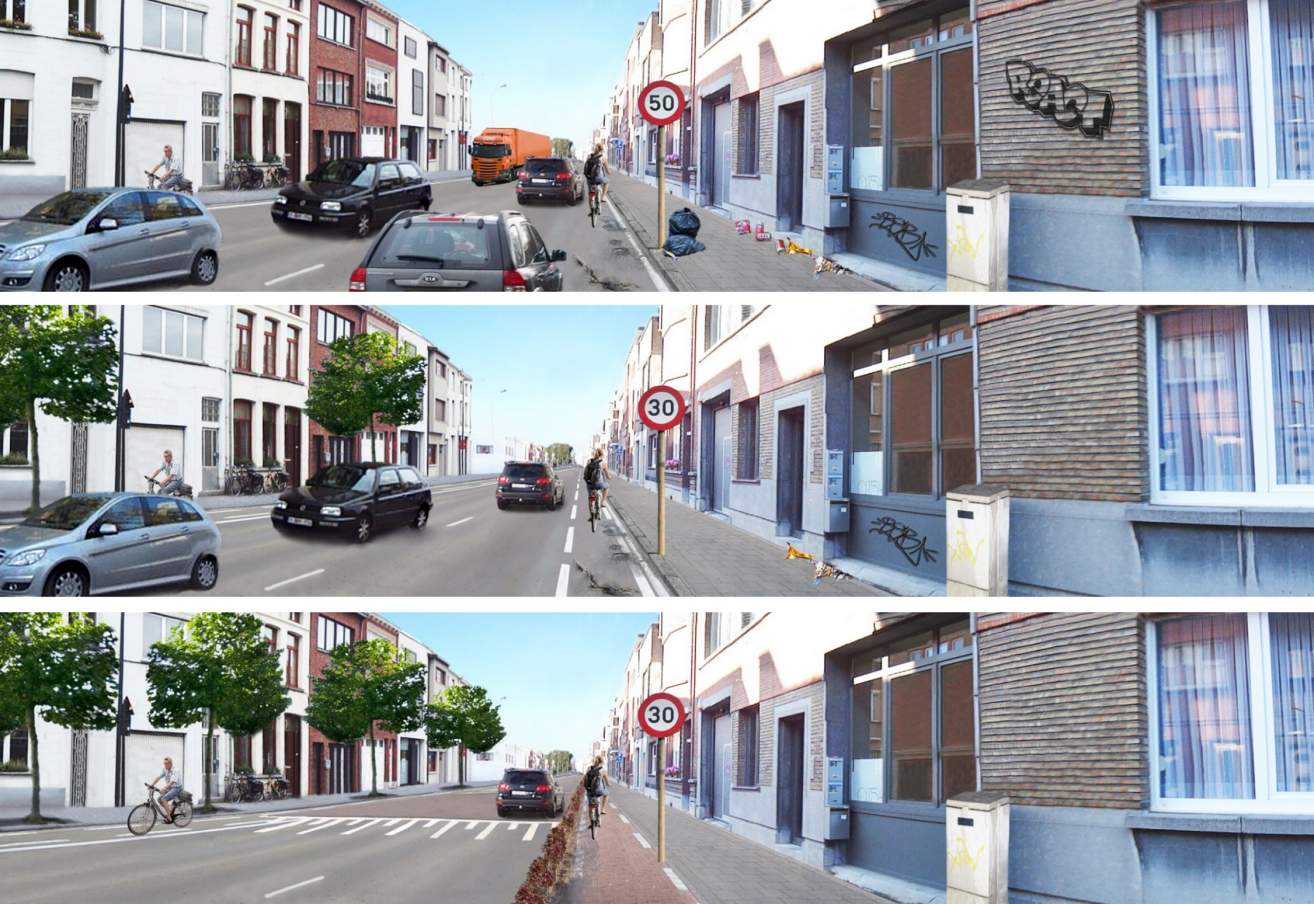
Examples of the manipulations in the photographs.

We kept some elements constant across all photographs to standardize the protocol, i.e. the general street setting (i.e. typical urban street), number of cyclists in the street, and good weather conditions. All photographs showed a cyclists’ point of view, to create the feeling that one is cycling in that street. Fig 1 shows an example of the performed manipulations, but an overview of all the conducted manipulations can be found in S1 Table. The first photograph shows the anticipated worst street to cycle along, the last photograph shows the anticipated best condition, and the second photograph shows an anticipated average street setting for cycling.

### Measures

Researchers developed online questionnaires using Sawtooth Software (SSI Web 8.3.10), one for children and one for parents. Both questionnaires had two parts. Children completed questions about their age, sex and socio-economic status by completing the Family Affluence Scale (results in a score ranging from 0 [lowest SES] to 10 [highest SES]) [40]. They indicated their physical activity levels in the last seven days (5 point scale; never/rarely–more than seven times) [41], reported their perceived cycling skills on a five-point scale (I think I can ride a bicycle properly; totally disagree-totally agree; intraclass coefficient [ICC] = 0.74) and how often they went to school by car or by bicycle (five point scale; never, rarely, now and then, often, always; ICC = 0.95 and 0.94 respectively). Developed for this study, these last three questions showed good one week test-retest reliability among a group of 45 children from fifth and sixth grade.

In the second part of the questionnaire, children completed a set of 12 choice-based conjoint (CBC) tasks. Conjoint analysis has participants choose among multiple ‘products’, in this case ‘streets’. Seeing streets that varied in levels (e.g. evenness of cycle path was shown as one of three levels; very uneven, moderately uneven and even cycle path) of the micro-scale environmental factors [37], participants needed to indicate which street they preferred most. CBC analysis benefits from the fact that participants do not have to choose between all possible combinations of attributes (micro-scale environmental factors) but the software assigns choice combinations randomly to each participant. Most CBC studies use verbal descriptions of the attribute levels of the products, but studies have successfully used photographs [34, 42].

Children were instructed to indicate which route they preferred to cycle along to their friend who lives 10 minutes by bike from the child’s residence, in good weather conditions. This instruction allows standardization for distance to the destination and weather conditions, which have been previously identified as potential determinants of children’s transportation cycling [43]. Before conducting the 12 randomly assigned choice-tasks, children completed 3 pre-exercises to get used to the differences in the photographs. A full profile design was used, which implies that the two photographs in each choice-task could differ on up to seven micro-environmental factors which also allows evaluation of the complete street, which is similar to a real-life decision making process.

In the parental questionnaire, parents first answered some questions about themselves, such as their age, highest level of education of the mother and father of the child and marital status. Then, they reported cycling behavior of their child and themselves based on the International Physical Activity Questionnaire (IPAQ; usual week [44]). They also answered ten questions about their perceptions of their neighborhood environment [45]. In the second part, parents completed a CBC task, which was similar to the children’s. Parents indicated which route they preferred *their child* to cycle along independently to visit a friend who lives ten minutes from their residence.

### Analysis

To compare children’s and parents’ preferences, the analyses considered only children whose parent also participated in the study. We used SPSS version 22 to calculate descriptive characteristics of the sample; and we used Sawtooth Software SSI Web (V8.3.10) to analyze the data from the CBC-tasks. We developed two models, one for the children and one for the parents. We estimated individual preferences (i.e. part-worth utility scores) through Hierarchical Bayes analysis, which is considered the best method for analyzing CBC data [46]. Part-worth utilities are considered as the preferences for an attribute level, which are considered as similar to regression coefficients in regression analyses [37]. Additionally, we calculated relative average importance percentages which demonstrate the maximum effect each micro-environmental factor has on the choice for a street for the total sample [37]. We calculated the average importance of each micro-scale environmental factor as follows: the range of the highest and the lowest part-worth utility within one micro-environmental factor, divided by the sum of the ranges of the part-worth utilities of the seven factors. Model fit is illustrated using the root likelihood (RLH), which ranges from zero to one, with a higher value indicating a better fit of the model.

Furthermore, we conducted latent class analysis in Sawtooth Software to examine whether the preference for a micro-environmental factor depicted in the photographs differed across subgroups, following the guidelines for conducting latent class analyses in Sawtooth (number of replications for each solution was set on 15) [47]. Subgroups were created based on having homogeneous preferences for the micro-environmental factors. The number of subgroups was determined based on increases in the goodness of fit of the models (expressed as log-likelihood and chi-square values). To examine differences in characteristics between the subgroups, we used chi-square analyses for categorical variables and MANOVA for continuous variables (Tukey post-hoc for homogeneous subgroups, Tham-Hane post-hoc for heterogeneous subgroups [48]). Differences among the subgroups of children were examined based on children’s characteristics, differences among the subgroups of parents were examined based on parental characteristics, age and gender of the child. Statistical significance was set at p = 0.05.

## Results

### Descriptive characteristics of the sample

2101 (response rate = 85.4%) children and 1284 (52.2%) parents completed the online questionnaire, resulting in 1232 child-parent pairs. Tables 2 and 3 show characteristics of the child-parent pairs. Children with participating parents were somewhat younger than those excluded from the analyses (10.52 vs 10.62 yrs; p<0.001), had a somewhat higher socio-economic status [based on FAS-score] (7.13 vs 6.93; p = 0.004), cycled more often to school [five point scale] (3.11 vs 2.88; p<0.001) and were less often driven to school (3.22 vs 3.38; p<0.01). We found no other differences for the child’s gender, physical activity levels and perceived cycling skills. More than 25% of the children did not cycle for transport in a usual week, while more than 50% of the parents indicated that they did not cycle for transport. Parents participating in the study were more educated compared to the general population (Flemish percentage of tertiary education = 43.3%, mothers in sample = 63.9%, fathers in sample = 48.8%). One third of the children indicated to be physically active more than 5 times a week, while 27% of the children indicated to be physically active only once or twice a week.

**Table 2:** Relative importance of the environmental factors within each subgroup among children.

	Total sample	Subgroup 1	Subgroup 2	Subgroup 3	Subgroup 4	*p-value*
***Segment Sizes***		28.90%	26.70%	23.60%	20.90%	
	n = 1232	n = 378	n = 307	n = 280	n = 266	
***Model fit (RLH)***	0.85	0.92	0.88	0.87	0.91	
***Relative attribute importance (%; 95% CI)***						
Type cycle path	**42.3 (41.8–42.8)**	**65.9 (65.4–66.4)**	**32.3 (31.5–33.0)**	**25.7 (24.8–26.5)**	**33.9 (33.4–34.3)**	
Speed limits	8.2 (8.0–8.4)	7.8 (7.3–8.2)	**14.2 (13.4–15.0)**	6.1 (5.6–6.6)	4.0 (3.7–4.3)	
Speed bump	2.4 (2.3–2.6)	2.0 (1.8–2.2)	3.3 (3.0–3.6)	3.6 (3.2–4.0)	2.6 (2.3–2.8)	
Vegetation	4.9 (4.5–5.4)	4.5 (4.2–4.8)	6.1 (5.6–6.5)	7.1 (6.5–7.7)	5.8 (5.4–6.1)	
Evenness of cycle path	**12.8 (12.0–13.6)**	6.5 (6.2–6.7)	5.9 (5.5–6.3)	**11.6 (11.1–12.1)**	**29.0 (28.5–29.5)**	
Maintenance	**15.0 (14.7–15.3)**	7.6. (7.3–8.0)	8.1 (7.8–8.5)	**35.1 (34.3–35.8)**	12.1 (11.8–12.4)	
Traffic density	**14.4 (14.0–14.8)**	5.7 (5.4–6.0)	**30.2 (29.2–31.2)**	10.8 (10.3–11.4)	12.7 (12.3–13.1)	
***Characteristics of the children***						
Sex (% boys)	49.6	53.2	52.8	46.8	43.8	*0*.*055*
Independent mobility (% not allowed to cycle on their own)	37.5	37.6	36.8	36.8	39	*0*.*948*
Good perceived cycling skills (% totally agree)	56.3	56.3	53.7	59.6	55.4	*0*.*099*
SES (/10)	7.1 ± 1.4	7.2 ± 1.4	7.1 ± 1.4	7.2 ± 1.4	7.0 ± 1.5	*0*.*268*
Age (yrs, Mean ± SD)	10.5 ± 0.6	10.6 ± 0.6	10.5 ± 0.6	10.5 ± 0.6	10.5 ± 0.6	*0*.*120*
Cycling per week (Minutes ± SD)	54.1 ± 60.9	62.3 ± 66.2^d^	51.2 ± 55.9	52.2 ± 64.4	46.7 ± 51.3^a^	*0*.*008*
Parents' cycling per week (Minutes ± SD)	47.0 ± 102.8	47.1 ± 85.6	57.6 ± 141.0^d^	46.2 ± 93.5	34.6 ± 79.1^b^	*0*.*070*
***Parental perceived neighborhood environment (5-point scale)***						
Amount of single unit houses	3.2 ± 1.3	3.4 ± 1.3^d^	3.1 ± 1.3	3.3 ± 1.3	3.1 ± 1.3^a^	*0*.*015*
Neighborhood traffic safety	3.6 ± 1.1	3.7 ± 1.1^c^	3.5 ± 1.0	3.4 ± 1.1^a^	3.7 ± 1.1	*0*.*013*
Neighborhood safety of crime	1.9 ± 0.9	2.0 ± 0.9	1.9 ± 0.9	2.0 ± 0.9	1.9 ± 0.9	*0*.*330*
Sufficient cycling infrastructure	2.7 ± 1.2	2.6 ± 1.2	2.7 ± 1.1	2.7 ± 1.1	2.7 ± 1.2	*0*.*729*
Good maintenance of cycling infrastructure	2.7 ± 1.2	2.7 ± 1.2	2.7 ± 1.1	2.7 ± 1.1	2.7 ± 1.1	*0*.*825*
Presence of vegetation	3.2 ± 1.1	3.2 ± 1.1	3.3 ± 1.0	3.1 ± 1.0	3.1 ± 1.1	*0*.*219*

^a^ significant difference with subgroup 1

^b^ significant difference with subgroup 2

^c^ significant difference with subgroup 3

^d ^significant difference with subgroup 4

**Table 3:** Relative importance of the environmental factors within each subgroup among parents.

	Total sample	Subgroup 1	Subgroup 2	Subgroup 3	Subgroup 4	*p-value*
***Segment Sizes***		47.1%	32.1%	12.4%	8.4%	
	n = 1232	n = 580	n = 395	n = 153	n = 104	
***Model fit (RLH)***	0.91	0.95	0.88	0.94	0.91	
***Relative attribute importance (%; 95% CI)***						
Type cycle path	**60.6 (59.9–61.2)**	**70.9 (70.4–71.4)**	**46.0 (45.2–46.8)**	**57.3 (56.5–58.1)**	**40.2 (39.3–41.2)**	
Speed limits	8.8 (8.5–9.0)	7.6 (7.4–7.9)	5.4 (4.9–5.9)	**23.3 (22.8–23.7)**	8.4 (7.6–9.2)	
Speed bump	3.9 (3.8–4.0)	3.9 (3.6–4.3)	4.3 (3.7–4.8)	5.3 (4.9–5.7)	2.9 (2.5–3.3)	
Vegetation	3.0 (2.9–3.1)	3.8 (3.6–4.1)	3.8 (3.4–4.1)	2.9 (2.6–3.1)	4.3 (3.8–4.8)	
Evenness of cycle path	7.2 (7.0–7.5)	4.3 (4.0–4.6)	**14.4 (13.4–15.3)**	3.7 (3.4–4.0)	8.6 (8.0–9.1)	
Maintenance	8.8 (8.4–9.1)	5.3 (5.1–5.5)	**17.9 (16.9–18.9)**	4.2 (3.8–4.6)	5.4 (4.9–5.9)	
Traffic density	7.8 (7.5–8.2)	4.2 (3.9–4.4)	8.2 (7.7–8.8)	3.4 (2.9–3.8)	**30.2 (29.1–31.4)**	
***Characteristics of the parents***						
Sex (% fathers)	22.7	23.1	21.0	24.2	25.0	*0*.*753*
SES (% tertairy education mother)	63.9	68.6	58.7	63.4	67.6	*0*.*140*
SES (% tertairy education father)	48.8	52.9	42.0	52.9	45.2	*0*.*023*
Childs' independent mobility (% allowed to cycle alone to school)	61.0	62.6	62.0	53.6	59.6	*0*.*221*
Childs' independent mobility (% allowed to cycle alone to other destinations)	64.3	65.9	65.8	54.2	65.4	*0*.*051*
Age (yrs, Mean ± SD)	41.9 ± 4.5	41.8 ± 4.3	41.5 ± 4.4^c^	42.5 ± 4.8	42.9 ± 5.8^a^	*0*.*013*
Cycling per week (Minutes ± SD)	47.0 ± 102.8	50.7 ± 93.0	35.4 ± 79.8	58.0 ± 164.6	54.0 ± 111.7	*0*.*045*
Childs' cycling per week (Minutes ± SD)	54.1 ± 60.9	53.0 ± 58.2	58.1 ± 67.2	49.0 ± 55.9	53.0 ± 58.0	*0*.*393*
						
***Perceived neighborhood environment (5-point scale)***						
Amount of single unit houses	3.2 ± 1.3	3.2 ± 1.2	3.2 ± 1.3	3.2 ± 1.3	3.2 ± 1.3	*0*.*938*
Neighborhood traffic safety	3.6 ± 1.1	3.6 ± 1.1	3.6 ± 1.1	3.6 ± 1.0	3.4 ± 1.2	*0*.*335*
Neighborhood safety of crime	1.9 ± 0.9	1.9 ± 0.8	2.0 ± 0.9	2.0 ± 1.0	1.91 ± 1.0	*0*.*392*
Sufficient cycling infrastructure	2.7 ± 1.2	2.7 ± 1.2	2.7 ± 1.2	2.7 ± 1.2	2.8 ± 1.1	*0*.*701*
Good maintenance of cycling infrastructure	2.7 ± 1.2	2.7 ± 1.2	2.6 ± 1.2	2.8 ± 1.2	2.9 ± 1.1	*0*.*090*
Neighborhood social environment	3.4 ± 0.9	3.5 ± 0.9	3.3 ± 0.9^b^	3.6 ± 1.0^a^	3.5 ± 1.0	*0*.*014*
Presence of vegetation	3.2 ± 1.1	3.2 ± 1.1	3.1 ± 1.0	3.3 ± 1.1	3.3 ± 1.1	*0*.*285*

^a^ significant difference with subgroup 2

^b^ significant difference with subgroup 3

^c ^significant difference with subgroup 4.

### Relative importance of the environmental factors in the total sample

**Fig 2:**
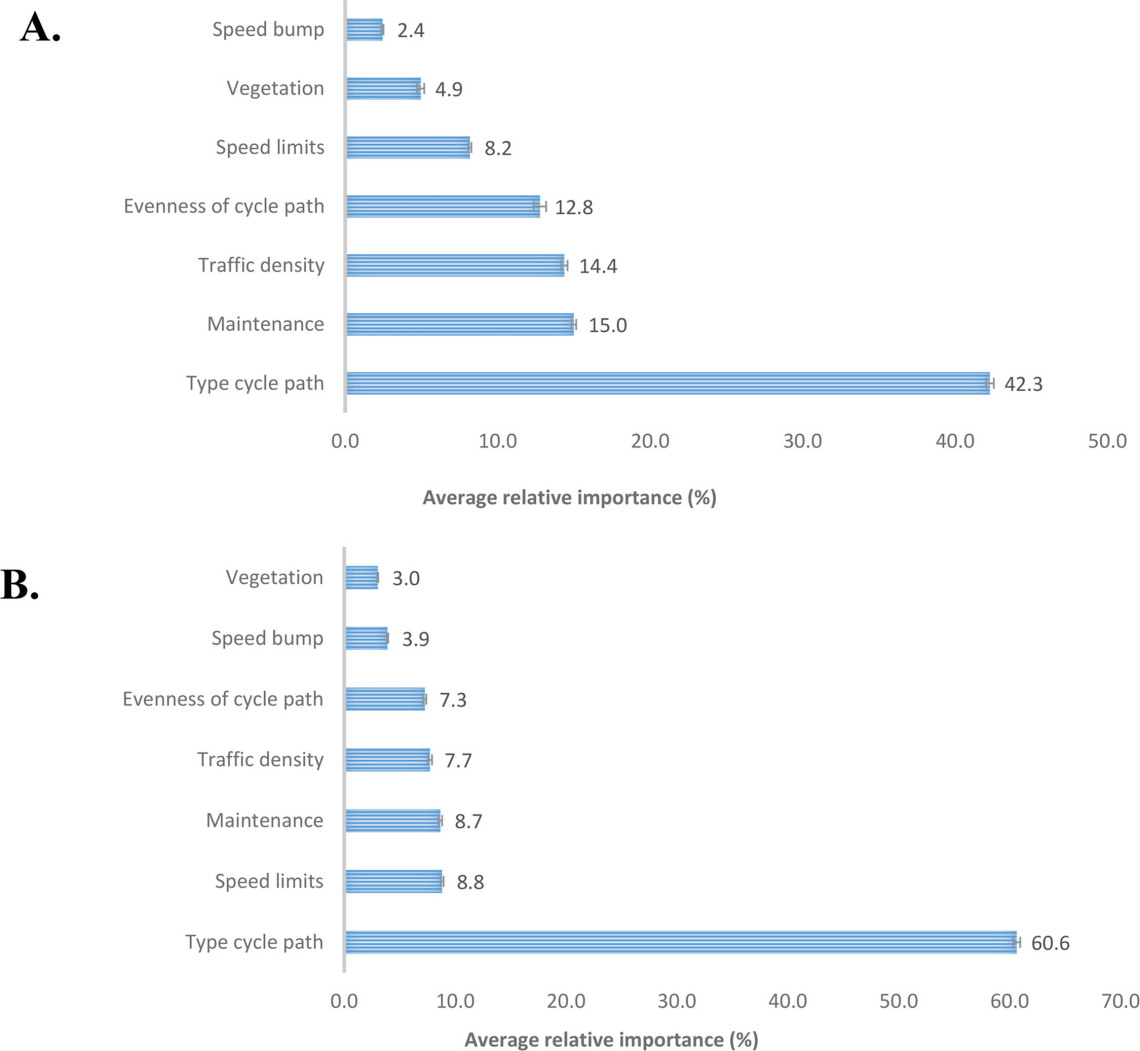
The relative importance and standard errors of each environmental factor for both children (A) and their parents (B).

Both children’s and their parents’ choices were mainly determined by the type of cycle path (see Fig 2, Tables 2 and 3). Among children, maintenance of the street and traffic density were the second most important factors, followed by evenness of the cycle path, speed limits, vegetation and presence of speed bumps. Among parents, the second most important factors were speed limits and maintenance, followed by traffic density and evenness of the cycle path, speed bumps and vegetation.

**Fig 3:**
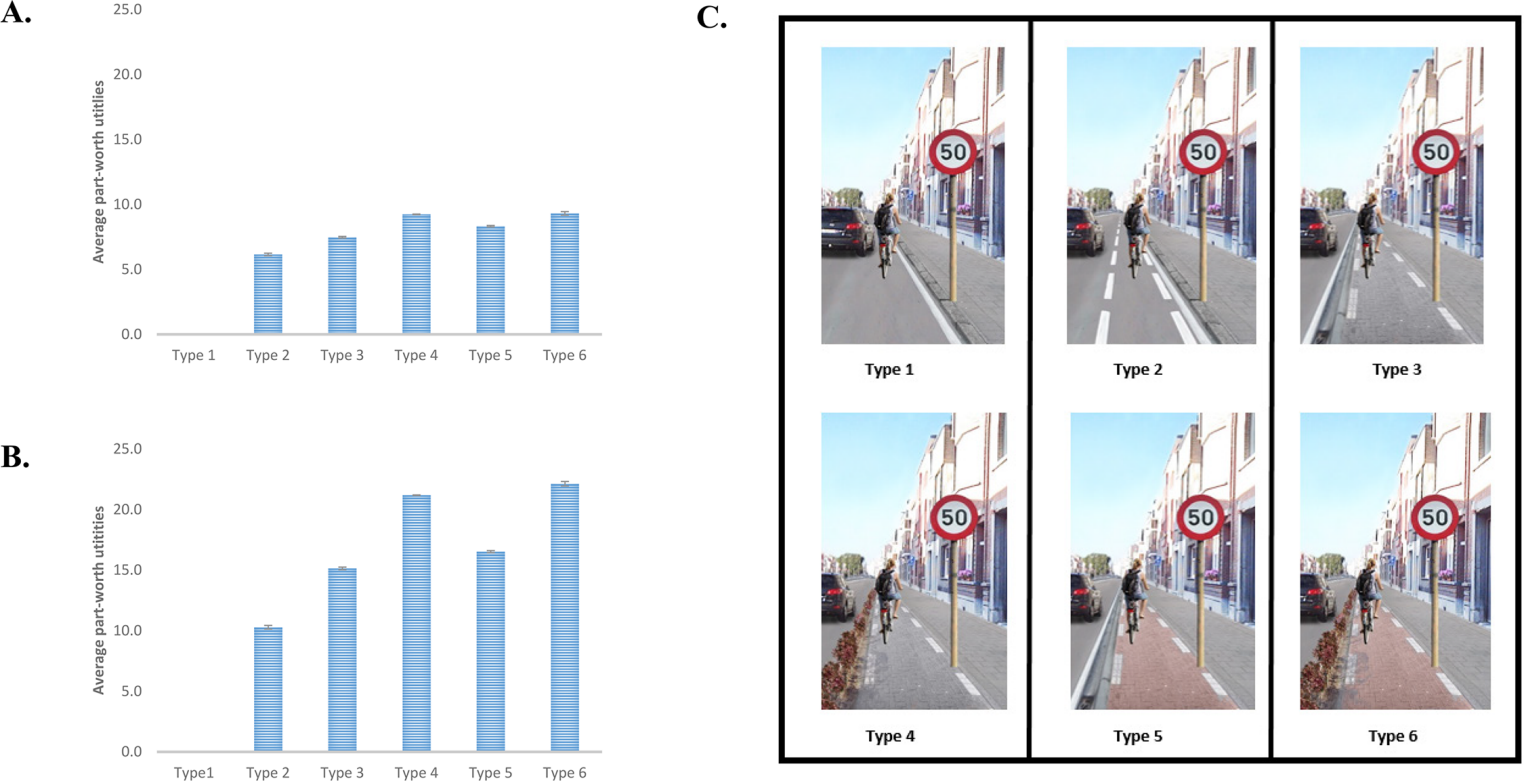
Part-worth utilities/preferences within type of cycle path among children (A) and their parents (B). Section C visually shows the different types of cycle path.

Furthermore, clear preferences were observed for a specific level within each environmental factor among children and their parents (see S6–S11 Tables). Within type of cycle path, children preferred to cycle on an elevated cycle path, separated from traffic with a hedge, and separated from the pedestrians with a difference in pavement color (part-worth utility = 9.3; 95% CI = 9.0–9.6; see Fig 3A and 3C). This type of cycle path was preferred to all other types of cycle path, except for an elevated cycle path separated from traffic with a hedge, but no additional color separation with the pedestrians (part-worth utility = 9.2; 95% CI = 9.2–9.3). An elevated red cycle path (part-worth utility = 8.3; 95% CI = 8.2–8.4) was preferred to an elevated cycle path (part-worth utility = 7.5; 95% CI = 7.3–7.6) and to an advisory cycle path (part-worth utility = 6.1; 95% CI = 6.0–6.3). The presence of any type of cycle path was preferred to no cycle path at all (part-worth utilities of all types of cycle path differed significantly from the reference category, i.e. having no cycle path).

For all other environmental factors (i.e. speed limit, presence of speed bump, evenness of cycle path, maintenance, vegetation and traffic density), all part-worth utilities significantly differed from each other and the anticipated best level was preferred within all different environmental factors (i.e. 30km/h, speedbump present, four trees, even cycle path, good maintenance and one car). Similar results emerged for parents, except they had a higher preference for an elevated red cycle path separated with a hedge (part-worth utility = 22.1; 95% CI = 21.8–22.5; see Fig 3B and 3C) compared to an elevated cycle path with a hedge with no additional separation with the pedestrians (21.2.; 95% CI = 21.1–21.2). All part-worth utilities of the other environmental factors differed significantly from each other, with a preference for the anticipated best level within each environmental factor (i.e. 30km/h, speedbump present, four trees, even cycle path, good maintenance and one car).

### Subgroup analysis children

Four different subgroups were identified, with specific preferences for some environmental factors as shown in Table 2 and in S1–S4 Tables. Within the largest group of children (subgroup 1), type of cycle path was by far the most important factor for route choice. Children in this group reported the highest cycling rates and reported the lowest neighborhood building density. Within the second group, type of cycle path was also the most important factor but traffic density was almost as important for route choice. Subgroup 2 consisted of children with parents who cycled most. Within subgroup 3, route choice was predominantly determined by maintenance of the street, followed by type of cycle path. This group lived in a neighborhood with highest perceived traffic safety. Finally, subgroup’s 4 most important factor for route choice was type of cycle path, but evenness of the cycle path was almost as important within this subgroup. Subgroup 4 was characterized with the lowest cycling rates for both children and parents, and lived within neighborhoods with the highest building density across the four subgroups. For 76.5% of the children (subgroup 1, 2 and 4), type of cycle path was the most important factor to determine preferred route choice.

### Subgroup analysis parents

Also among parents, there were four subgroups with specific preferences for environmental factors (see Table 3 and S6–S9 Tables). Across the four subgroups, type of cycle path was of highest importance. Differences were observed for the second most important factors between the subgroups. Within the first subgroup, parents’ route choice was predominantly determined by type of cycle path. In this group, there were more fathers who obtained a tertiary education, compared to subgroup two and four. In the second subgroup, maintenance and evenness of cycle path were important factors next to the type of cycle path. This group had the lowest proportion of tertiary educated parents. Additionally, the children from the parents within this group cycled most, while the parents themselves cycled least among the four groups. In the third subgroup, type of cycle path was by far the most important factor, followed by evenness of the cycle path. These parents were those with the highest cycling rates, the best perceived social neighborhood, but almost half of them restricted their child to cycle independently to school or to other destinations within the neighborhood which is more than within the three other subgroups. Finally, within the fourth subgroup type of cycle path was the most important factor, but traffic density emerged to be important as well. These parents were somewhat older compared to the parents from the other subgroups and perceived their neighborhood cycling infrastructure as well maintained. Children’s age (MANOVA F = 1.499, p = 0.213) and gender (chi-square;χ^2^ = 0.506, *p* = 0.918) did not significantly differ across the four subgroups of parents.

## Discussion

This study examined the relative importance of seven micro-scale environmental factors which were hypothesized to influence the perceived supportiveness of a street for children’s transportation cycling. Qualitative research [24] had identified the seven elements as relevant, and the present study demonstrated quantitative effects. This study highlighted the importance of cycling infrastructure to increase the supportiveness of a street for children’s transportation cycling. The choices of children and their parents were predominantly influenced by the type of cycling infrastructure, which comprised mainly separation from other road users (see Fig 3), i.e. motorized traffic on the road, and pedestrians on the pavement. Clear preferences were observed for a separation with motorized traffic by a hedge, rather than no separation or separation by lines or a curb among both children and their parents, which is consistent with findings from our previous pilot study [34].

Furthermore, an additional separation from pedestrians by differences in pavement color was also preferred. Differences in pavement color can be considered as a good tool to separate walkers and cyclists. A previous study examined the effect of bollards that separated walking and cycling paths on the supportiveness for transportation cycling among mid-aged adults, but these were disliked as it limits the swerving alternatives for cyclists [31]. As resources are most often limited for changing the physical environment, it is important to invest in the environmental factors which are likely to have most effect on cycling behavior. Since the type of cycle path was the most important factor among 76% of the children and all parents, it is likely that investments in creating dedicated cycling infrastructure would be most efficient to create cycling-friendly environments. Cycling infrastructure can increase safety objectively and subjectively [49, 50], especially when it is separated from motorized traffic [51].

Children and their parents had some minor differences on the importance of the other micro-scale environmental factors. Although choices by children and parents agreed on the importance of type of cycle infrastructure, and the limited importance of vegetation and speed bump, they differed in that children gave more importance to traffic density, while among parents, traffic speed was of greater importance. It is remarkable that for both children and parents the general maintenance of the street had a considerable influence on their route choice preferences. Previous research has identified maintenance as a potentially important factor, as individuals prefer to be active in places they perceive as aesthetically appealing [22], but it was not hypothesized to be the second and third most important factor for children and their parents, respectively. Multiple studies showed that poor maintenance of the streets is a physical cue to social disorder [52]. It raises the fear of crime, and as a results, one could expect them to affect route choice for children’s cycling. A recent study examined the effect of physical disorder, such as well-maintained pavements, litter, vegetation in cracks, building maintenance etc., on children’s walking behavior, and concluded that the presence of a physical disorder reduced the likelihood of choosing a street for walking among both children and their parents [33]. As parents’ main focus was expected to be on safety-related elements [23], it could be that the presence of litter and graffiti was perceived as an indicator of low social neighborhood safety (from crime) rather than as aesthetically unpleasant [53, 54]. Finally, evenness of the cycle path was in our previous pilot study identified as the most important factor for children [34], while in the current study evenness of the cycle path was the third most important factor. In the pilot study, maintenance of the street and traffic density were kept constant and this study shows that these factors are more decisive on children’s route choice compared to evenness of the cycle path. Evenness relates more to comfort, which is subordinate to safety (traffic density and type of cycle path) and aesthetics (maintenance) for children.

Furthermore, both parents and children preferred 30km/h compared to 50 km/h, an even cycle path compared to a moderately or very uneven cycle path, presence of a speed bump, presence of vegetation compared to some or no vegetation, and low traffic density compared to higher traffic density with presence of trucks. These results indicate that future changes in the physical environment may benefit from improvements in the micro-scale environmental factors examined to increase the supportiveness for transportation cycling. If these findings hold, they suggest that communities can make relatively inexpensive and easy changes to the environment in order to increase children’s transportation cycling. These micro-scale environmental factors are the responsibility of local government, which means that micro-scale environmental changes can be conducted more rapidly compared to macro-scale factors such as the street connectivity [55].

When examining the subgroup analyses among both children and the parents, it was noticeable that there were subgroups that differed in preferences for specific environmental factors, but there were not many differences in characteristics between these subgroups (see Tables 2 and 3). The factors that were hypothesized to be associated with different preferences could only explain some differences between the groups. It could be that there are other individual characteristics, not included in this study that can explain the differences in individual preferences. For example, more information about cycling experience, such as fall history, might explain individual preferences. However, Stamps and colleagues found high correlation between environmental preferences across different demographic subgroups, but differences were observed regarding preferences among children and adults, which our study results also confirm [56]. For most participants (76.5% of the children and all parents), the type of cycle path appeared to be the most important factor. Previous studies consistently indicated that lack of infrastructure and low (perceived) safety are important barriers for children’s active transport, including cycling [20, 23]. Changing the physical environment by installing cycle paths, well-separated from motorized traffic, with an even surface and good street maintenance, low motorized traffic speed and low traffic density might induce a change in both the objective and perceived safety of that street, and therefore be most successful in promoting cycling for transport among children. We call for observations of behavior in real environments to test the validity of the present findings.

### Strengths and limitations

The present study heeded the call for studies of context-specific influences on cycling behavior [57], by leading the way in examining the relative importance of many physical environmental elements to a specific behavior, i.e. transportation cycling among children. In response to a lack of research on children’s environmental preferences for cycling [24, 58], the present study not only examined children’s preferences but it also used stated preference methods, typically used for adult cyclists [59] but for only one environmental factor, i.e. on bicycle infrastructure [60–62]. Additionally, the use of manipulated photographs integrated within a choice-based conjoint task addresses the shortcomings of the use of questionnaires [27, 28] and enables the examination of potentially causal relations between physical environmental factors and the supportiveness of a street for transportation cycling. Finally, a large sample of matched child-parent pairs participated in the study, which ensures that a wide variety of individuals was reached and which allows comparison between children’s and parents’ preferences, as we know that perceptions of both parents and children are important for deciding whether to cycle or not.

We acknowledge some caveats. The present research asked participants to indicate which route (displayed in photographs) they prefer to cycle along. We call for research that examines the degree to which changing these micro-scale environmental factors affects children’s cycling for transport. In light of possible selectivity in the sample (participants with computer at home, high proportion of mothers), researchers might seek a broader and more diverse sample of respondents.

## Conclusions

This study highlights the importance of micro-scale environmental factors on creating environments that are supportive for children’s transportation cycling. For both children and their parents, it was found that having any dedicated place to cycle (cycle path separated from motorized traffic by at least lines, or better, a curb or a hedge) appeared to be the most important factor to create a supportive environment for children’s cycling. In order to promote children’s transportation cycling, structural changes within the neighborhood might be most effective when having a clearly separated cycle path, separated from traffic with a hedge and being separated from the sidewalk with some color. These findings must be confirmed by on-site experimental research. If the results can be confirmed and implemented, the present findings can improve the quality of streets, increase cycling, improve health, reduce traffic congestion and improve quality of life for millions of people.

## Supporting Information

S1 Table Visual tool of the conducted manipulations in the photographs. (DOCX)

S2 Table part-worth utilities within children’s subgroup 1. (DOCX)

S3 Table part-worth utilities within children’s subgroup 2. (DOCX)

S4 Table part-worth utilities within children’s subgroup 3. (DOCX)

S5 Table part-worth utilities within children’s subgroup 4. (DOCX)

S6 Table part-worth utilities within the total sample of children. (DOCX)

S7 Table part-worth utilities within parents’ subgroup 1. (DOCX)

S8 Table part-worth utilities within parents’ subgroup 2. (DOCX)

S9 Table part-worth utilities within parents’ subgroup 3. (DOCX)

S10 Table part-worth utilities within parents’ subgroup 4. (DOCX)

S11 Table part-worth utilities within the total sample of parents. (DOCX)
