# Supplementary material for: Creating Cycling-Friendly Environments for Children: Which Micro-Scale Factors Are Most Important? An Experimental Study Using Manipulated Photographs
Source: PLoS One. 2015 Dec 1;10(12):e0143302. doi: 10.1371/journal.pone.0143302 (PMC4666668; doi:10.1371/journal.pone.0143302)
Supplement: S1 Table — (DOCX) [file pone.0143302.s001.docx]

| Type of cycle path | 1. No cycle path 2. Cycle path separated from traffic with lines, not separated from walking path (advisory cycle path) 3. Cycle path separated from traffic with a curb, not separated from walking path 4. Cycle path separated from traffic with a hedge, not separated from walking path 5. Cycle path separated from traffic with a curb, separated from walking path by colour 6. Cycle path separated from traffic with a hedge, separated from walking path by colour | 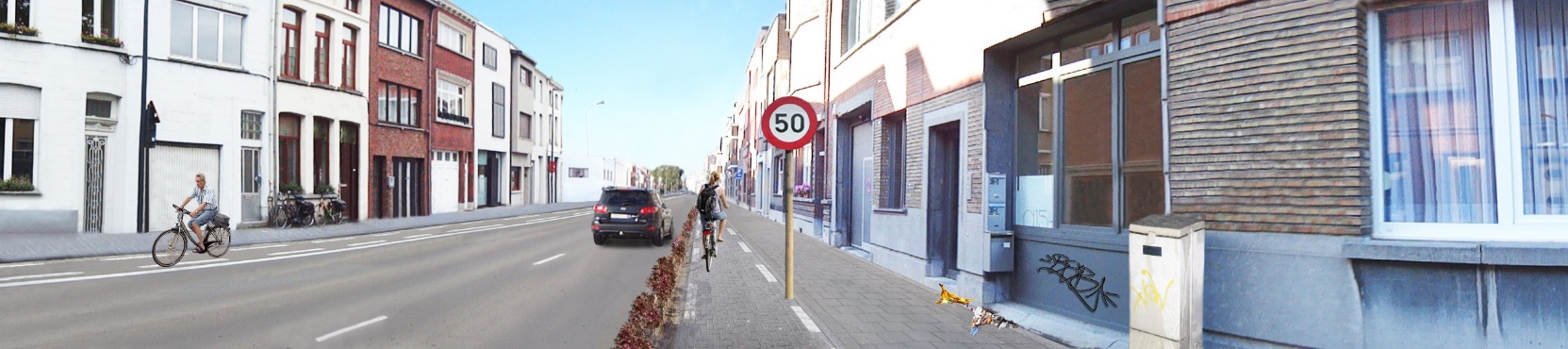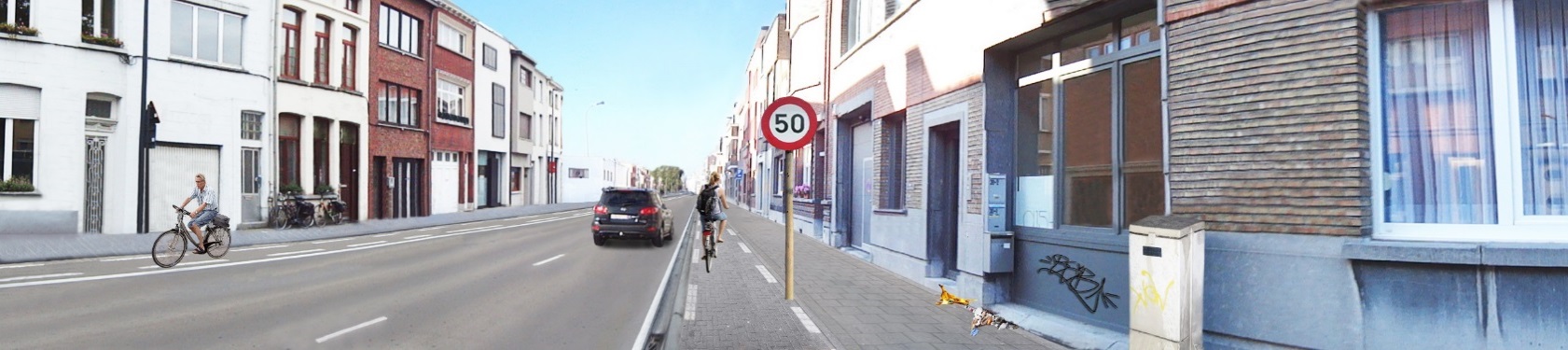 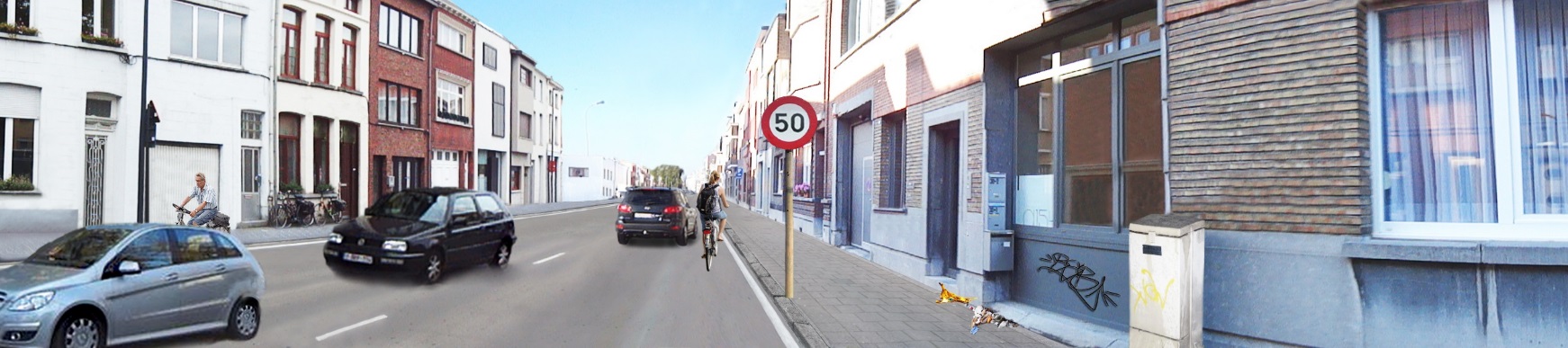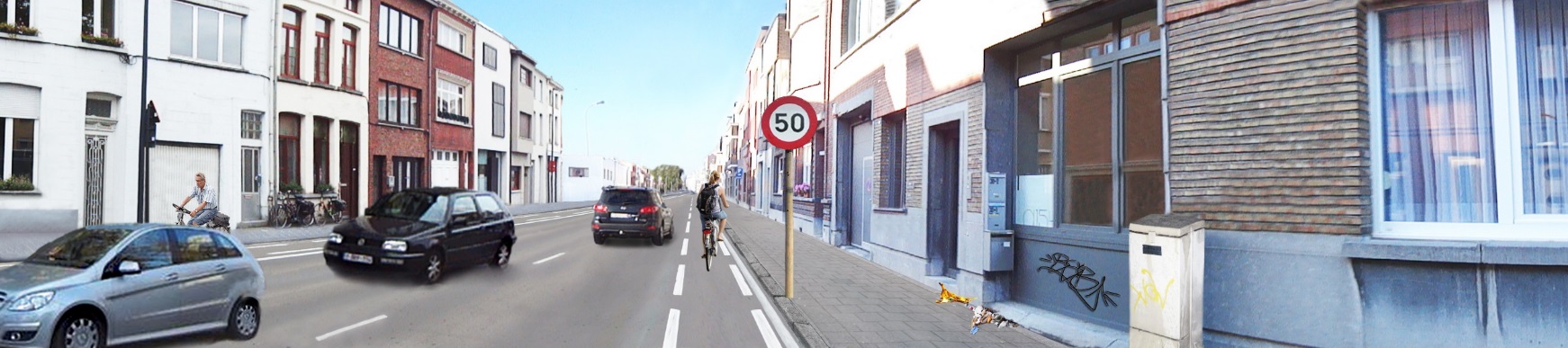   1. 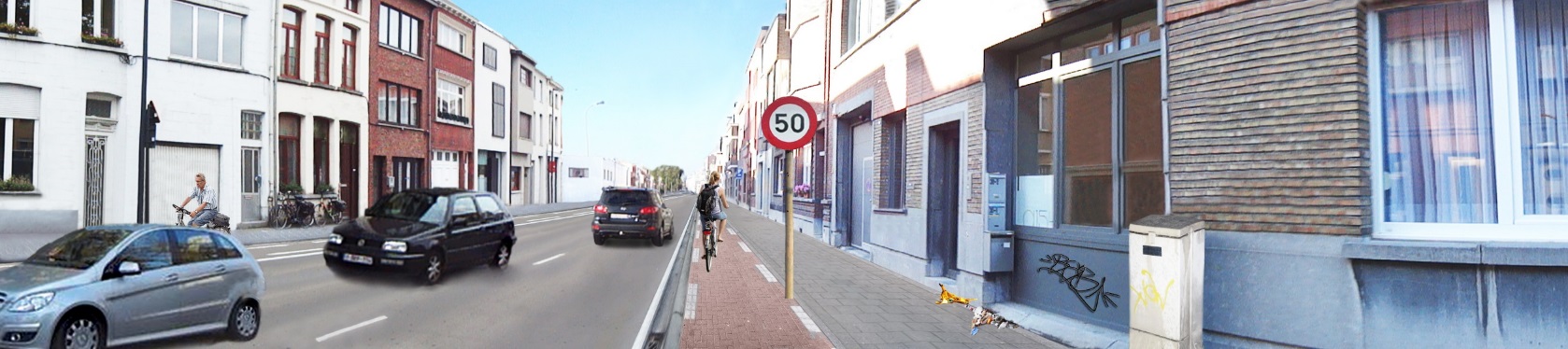 (2) (3)   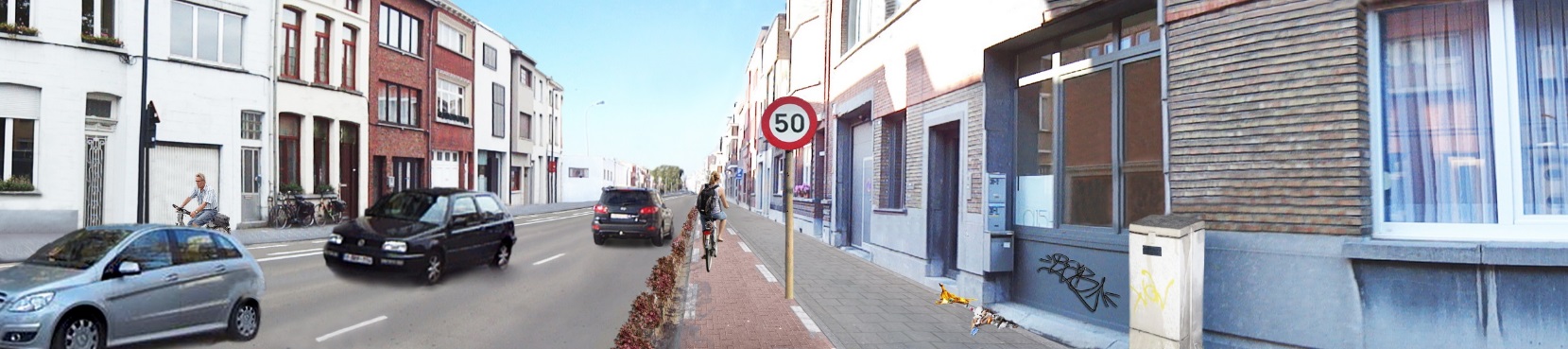  (4) (5) (6) |
| --- | --- | --- |
| Evenness of cycle path | 1. Very uneven 2. Moderately uneven 3. Even | 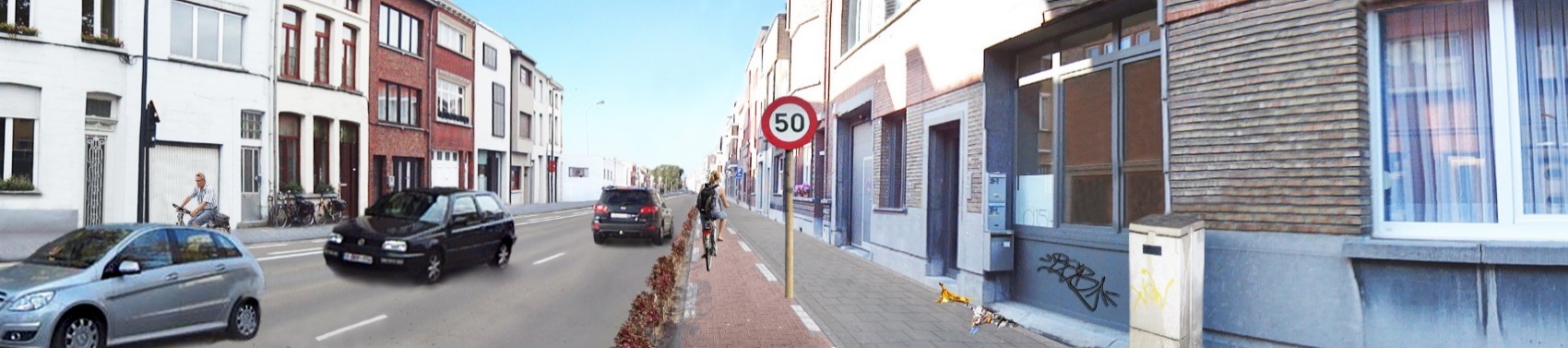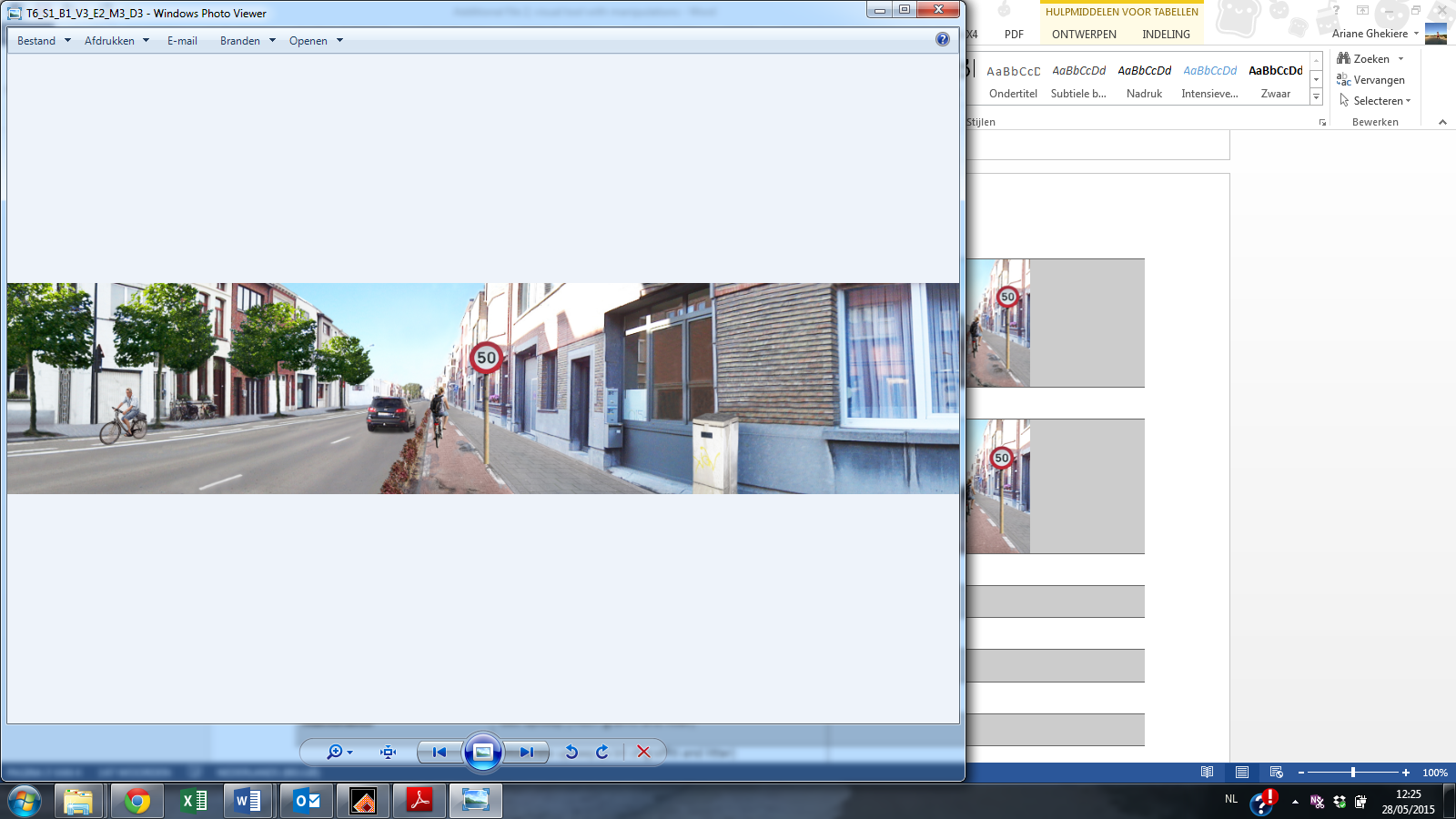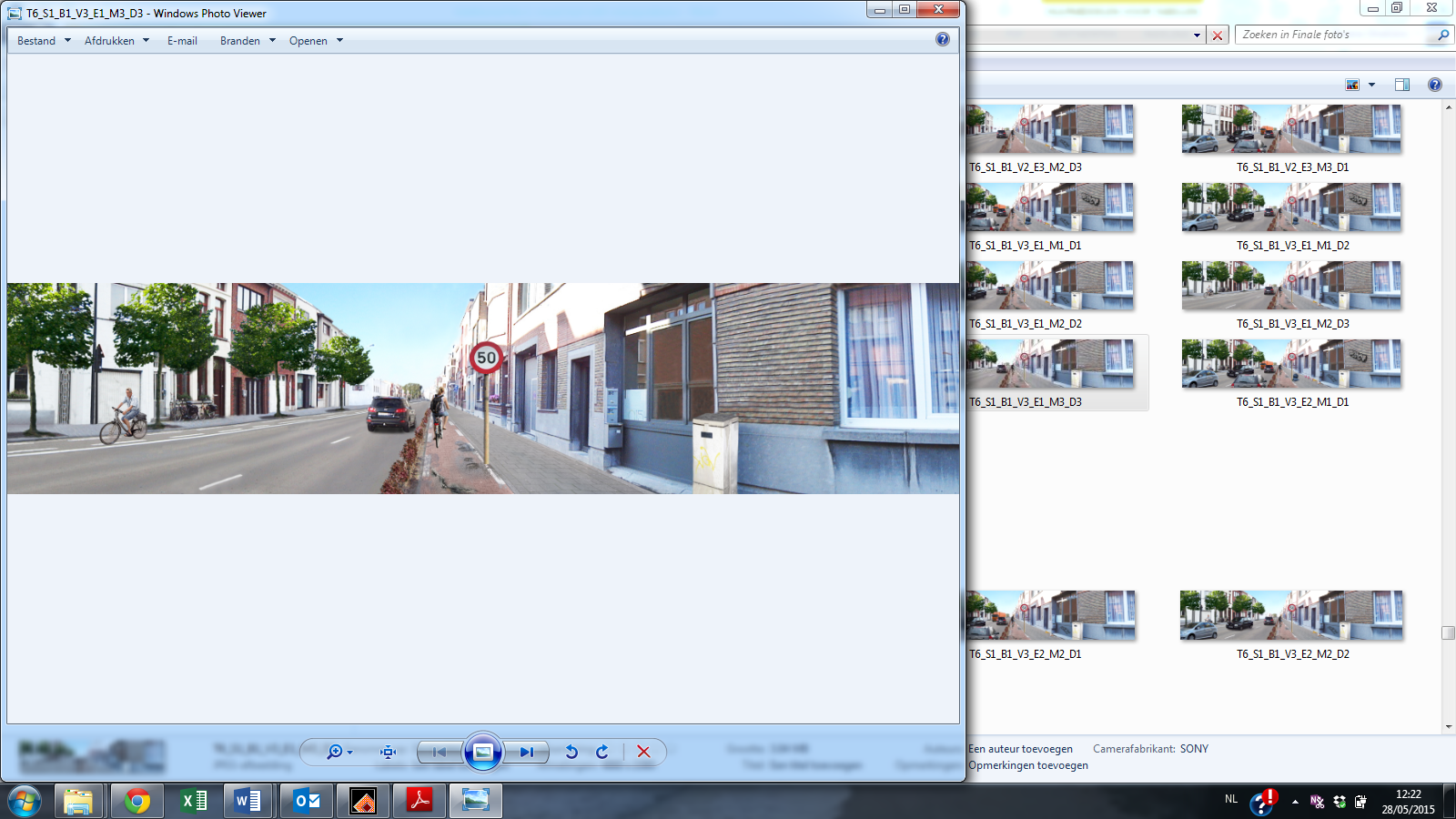     1. (2) (3) |
| Traffic speed | 1. 50 km/h 2. 30 km/h | 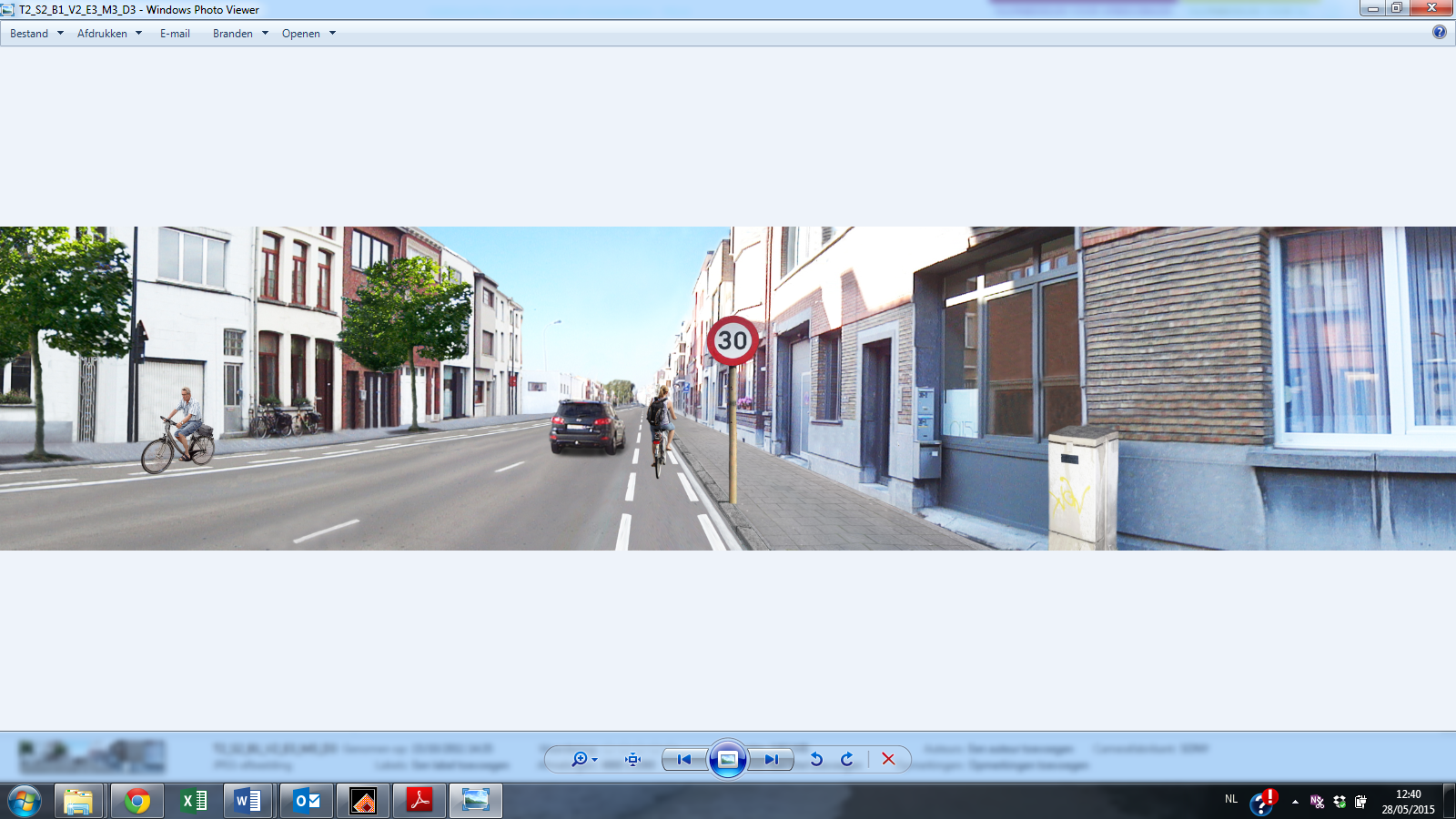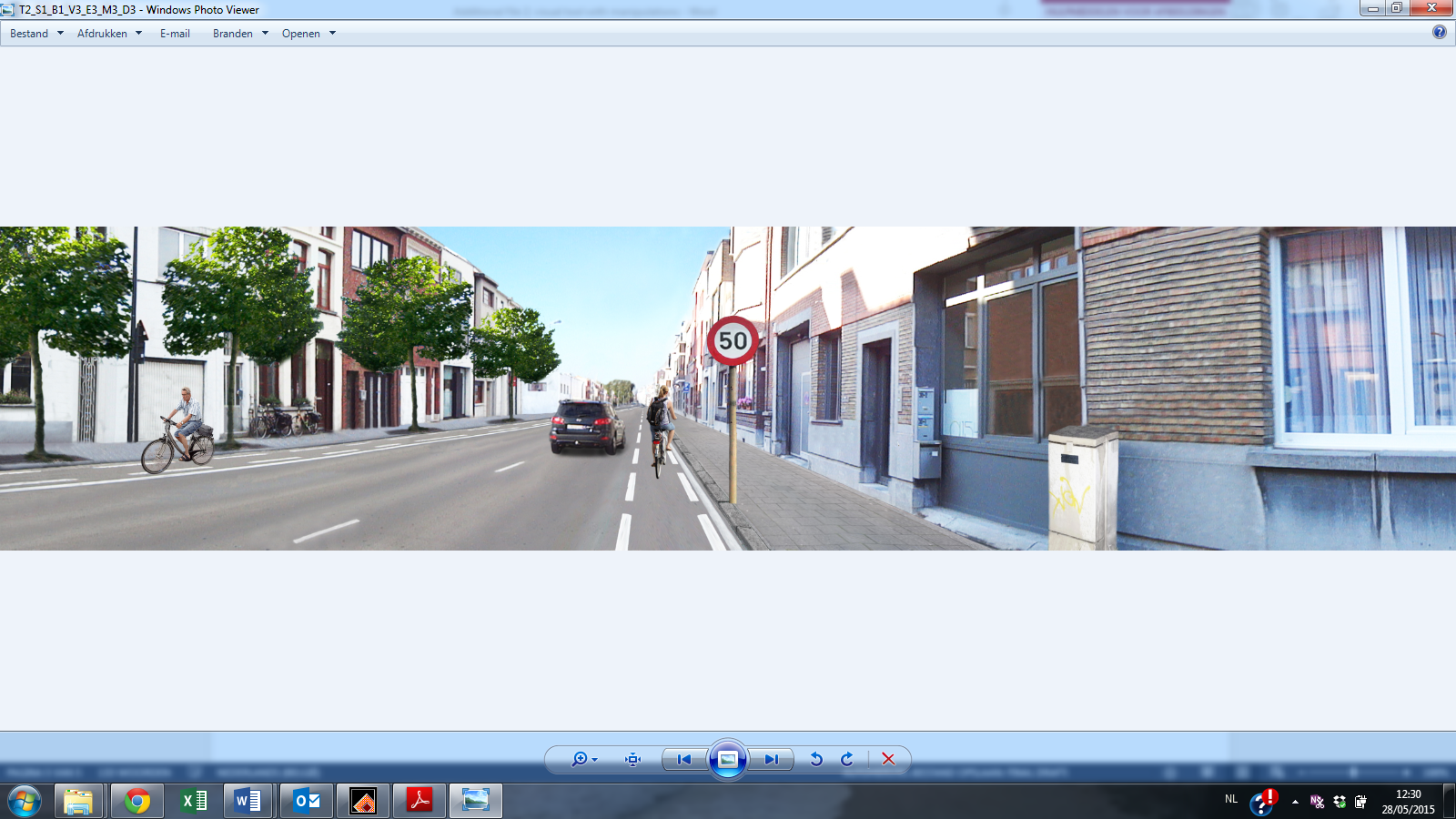     1. (2) |
| Vegetation | 1. No trees 2. Two trees 3. Four trees | 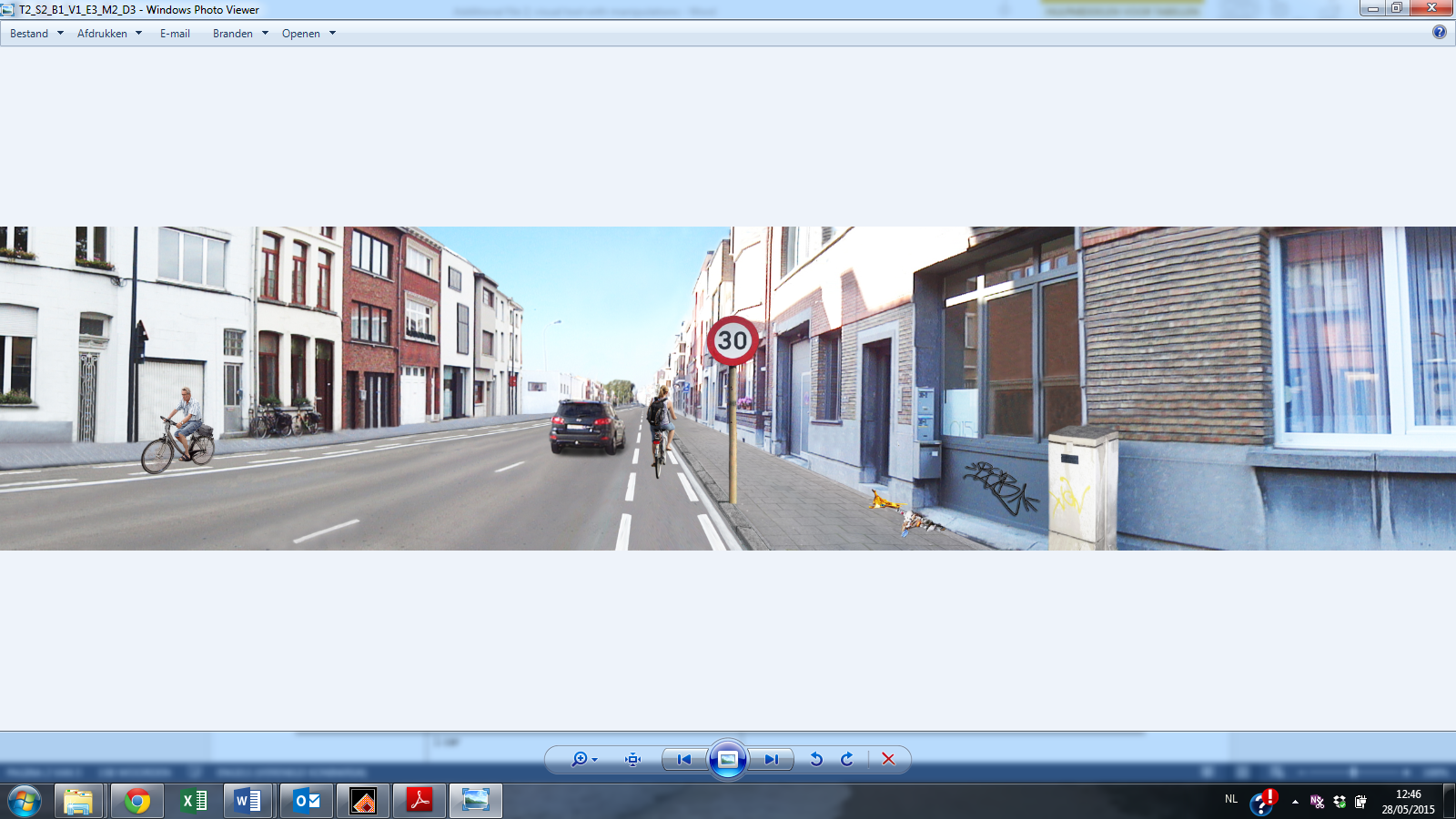      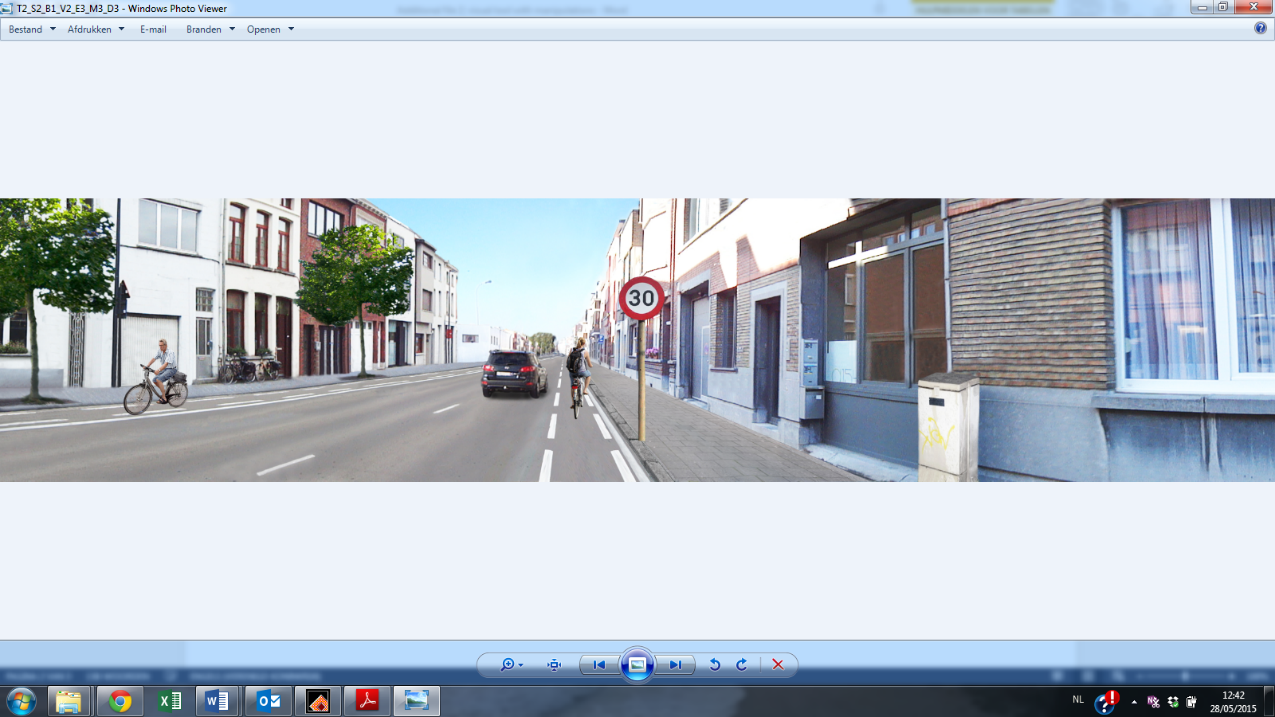  (2)  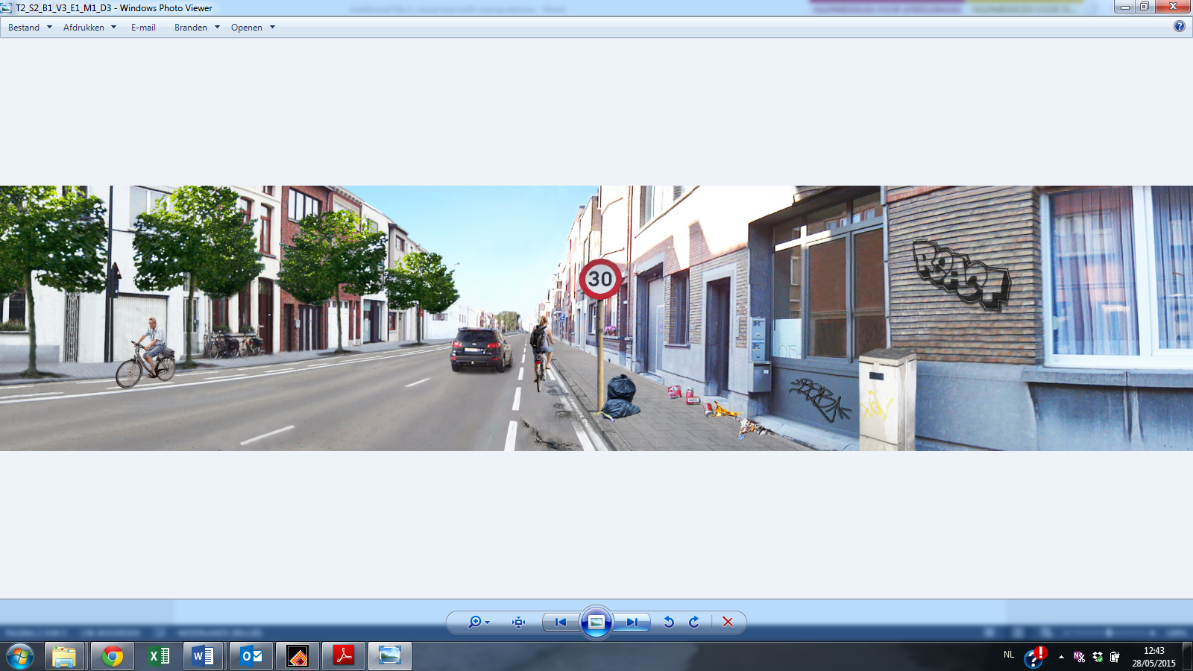  (3) |
| Maintenance | 1. Bad upkeep (much graffiti and litter) 2. Moderate upkeep (a bit of graffiti and litter) 3. Good upkeep (no graffiti or litter) | 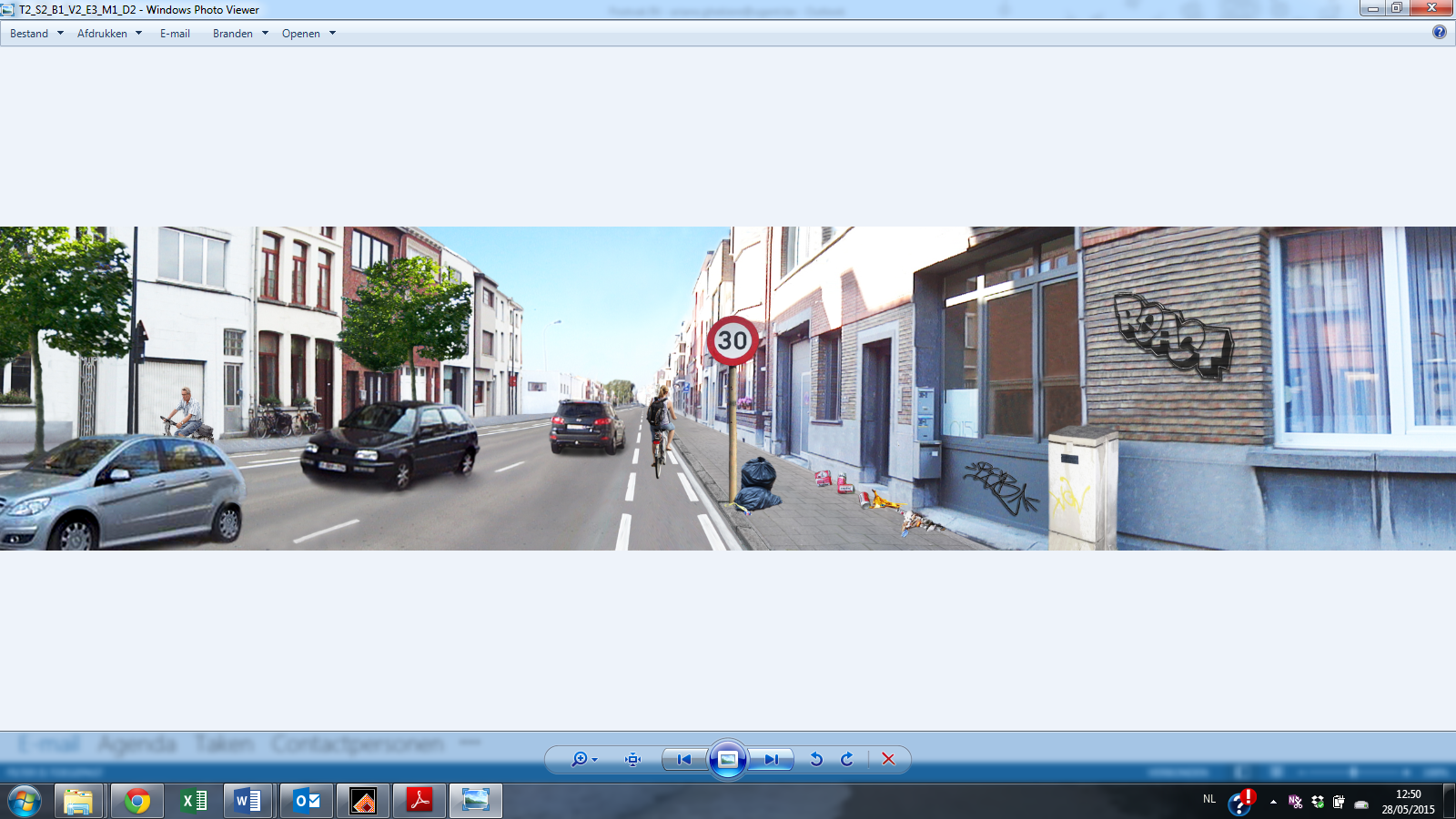   1. 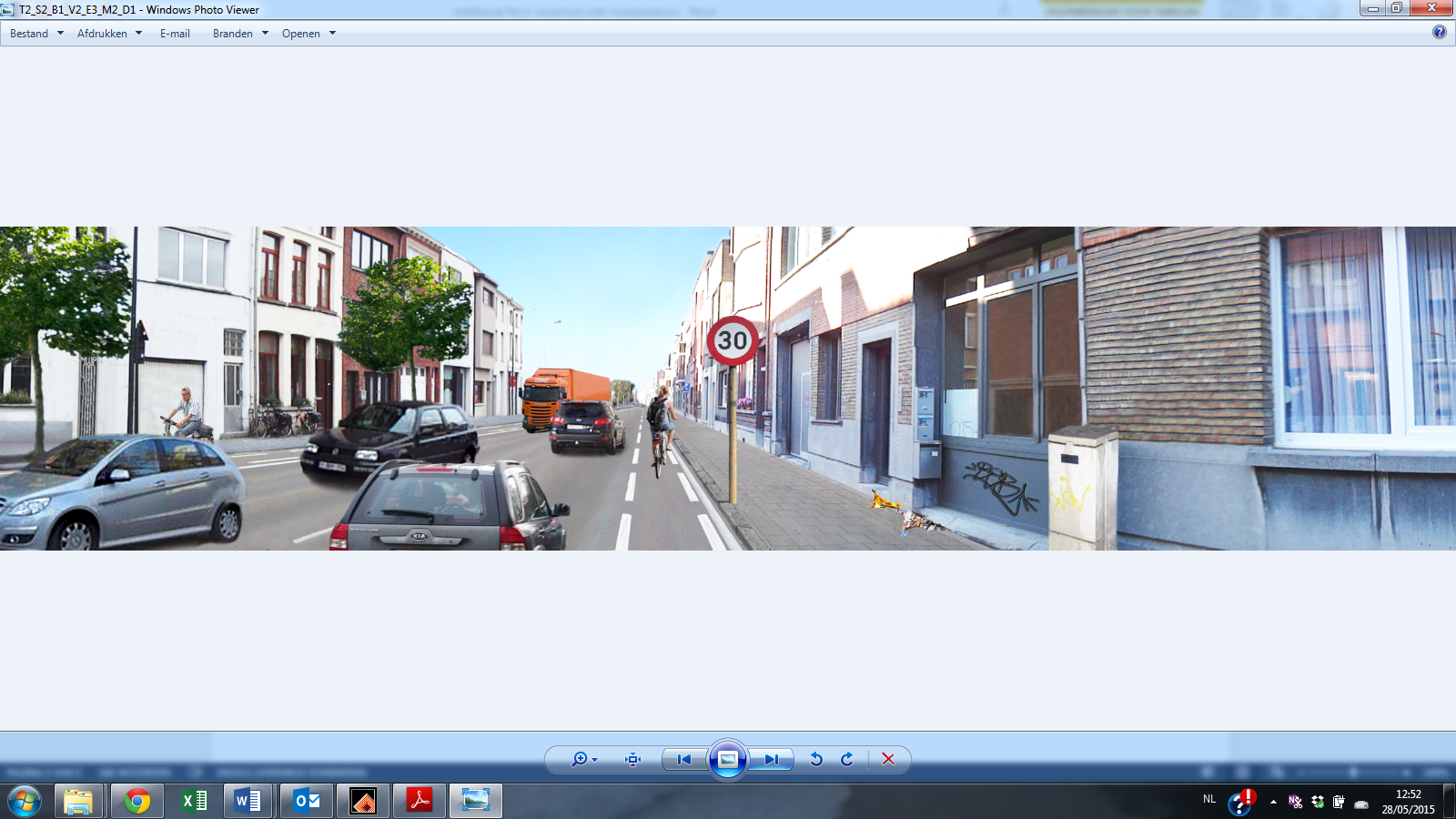 2. 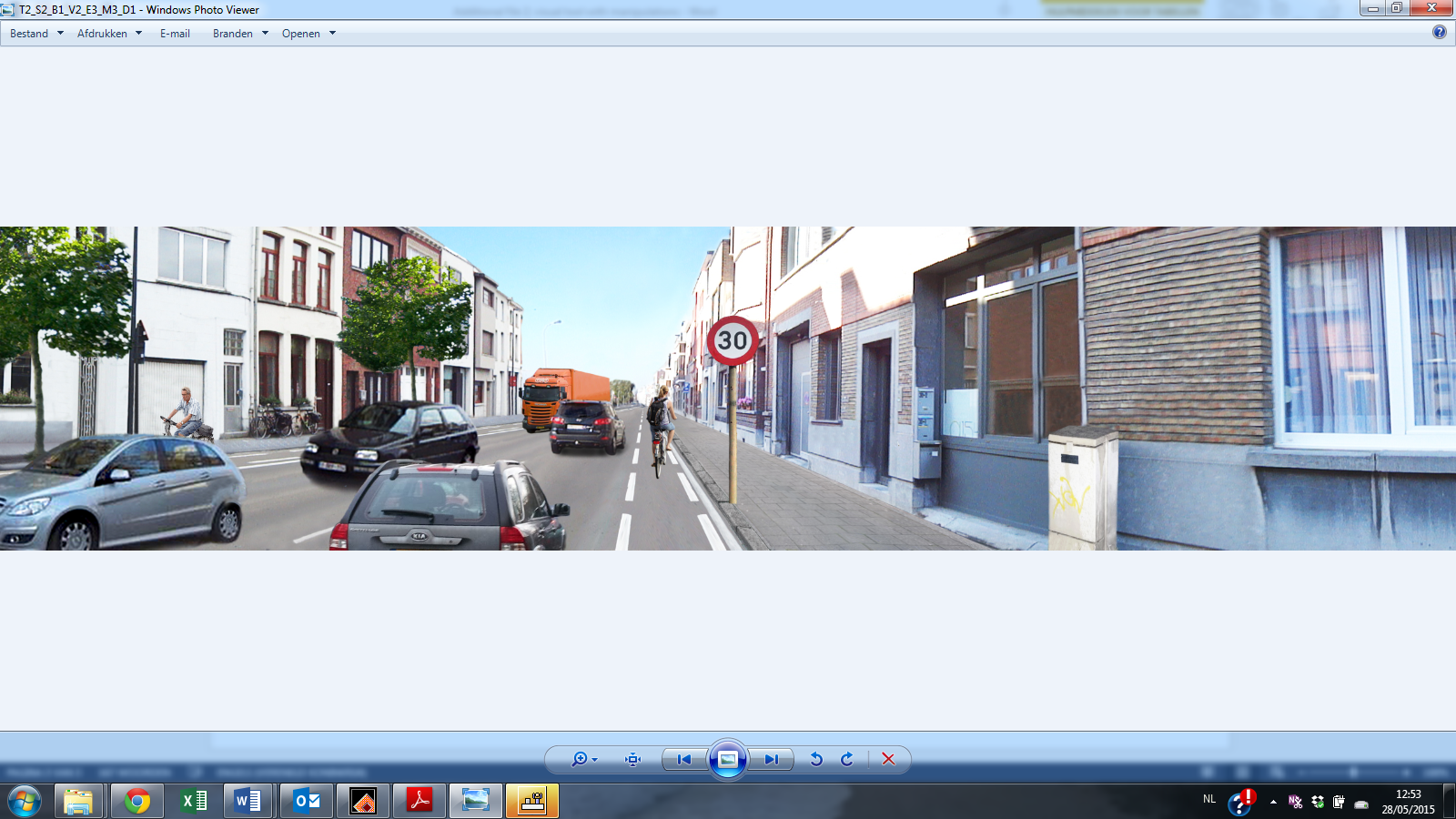 |
| Traffic density | 1. 4 cars + truck 2. 3 cars 3. 1 car | 1. 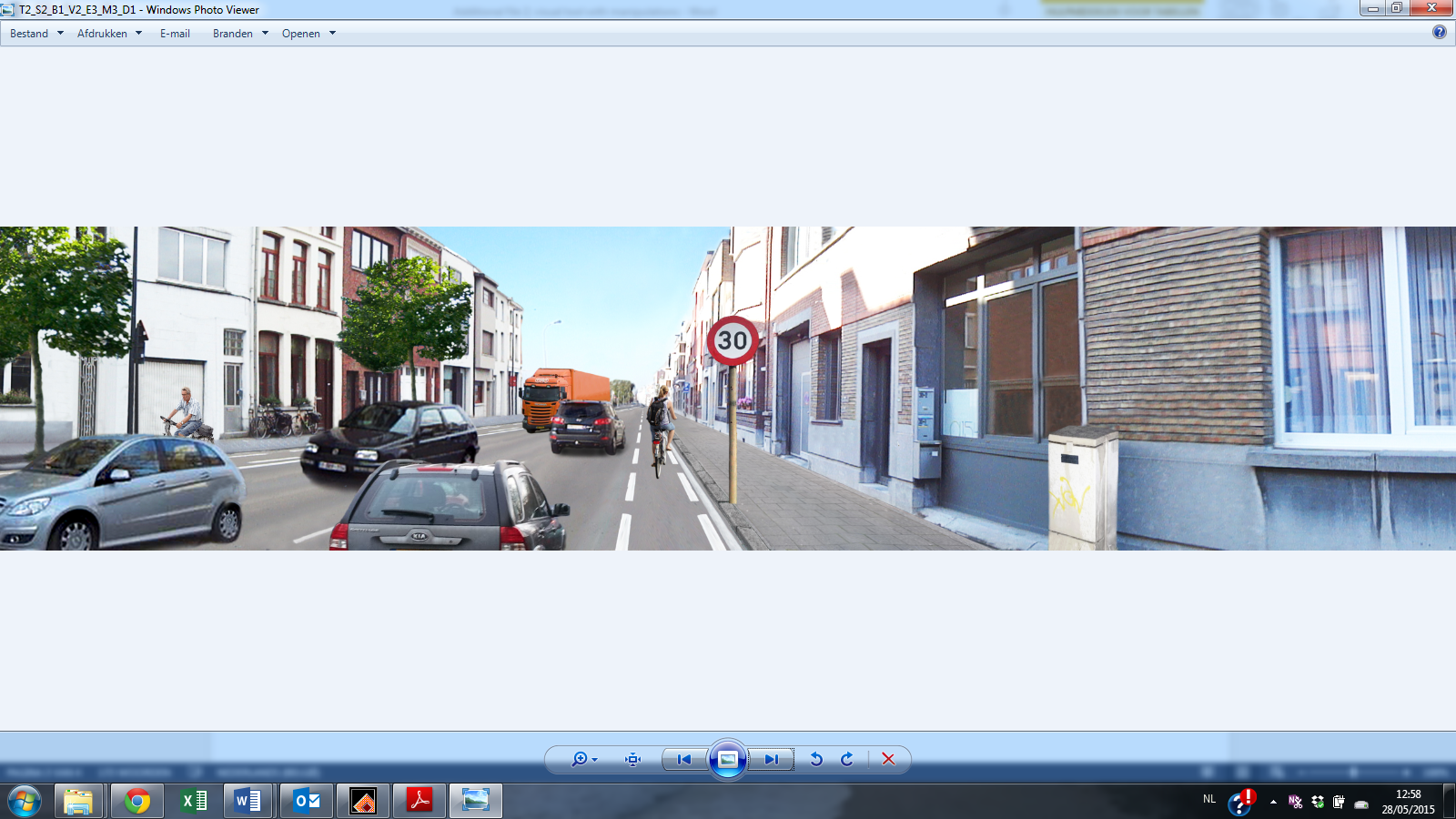 2. 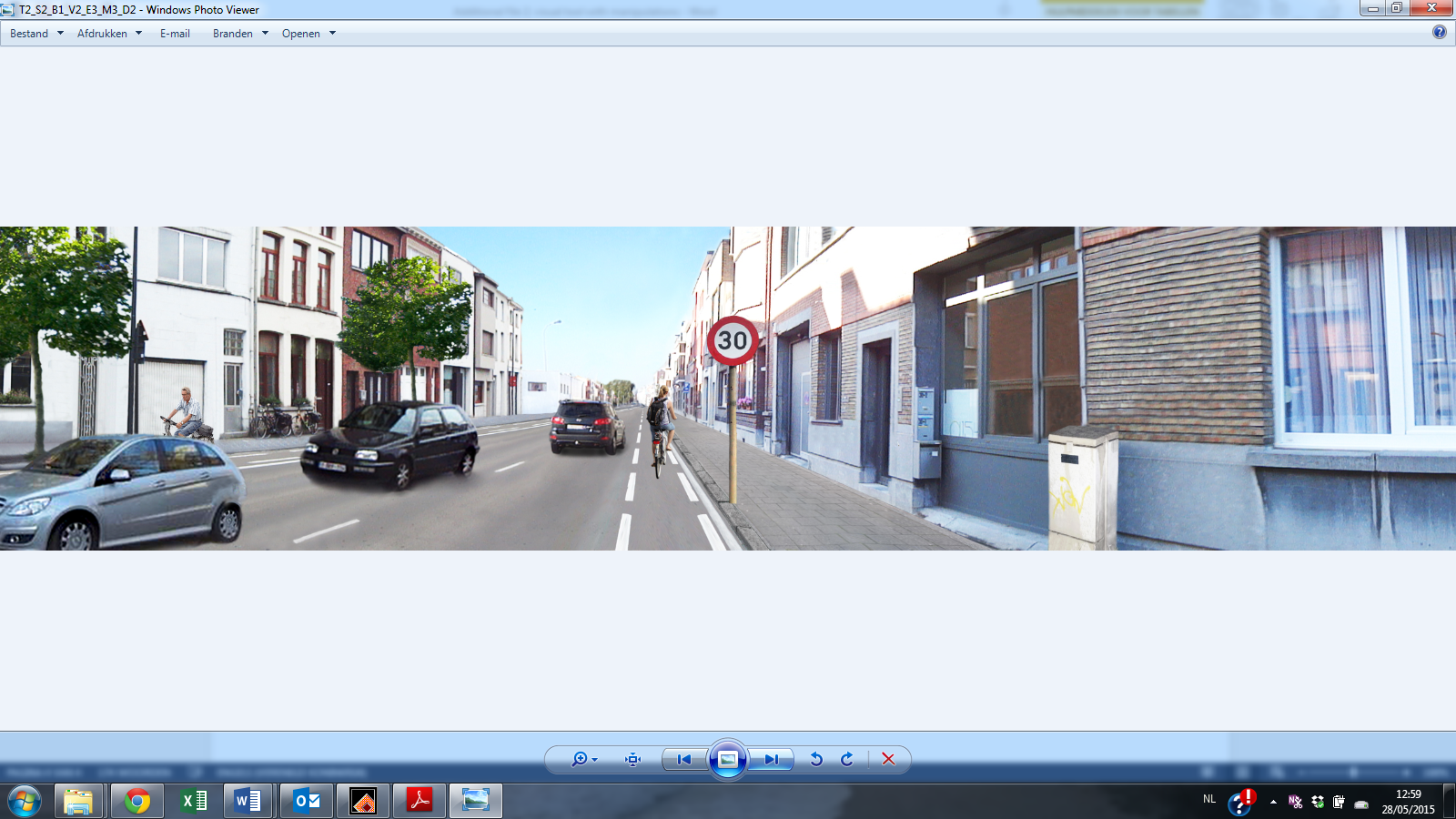 3. 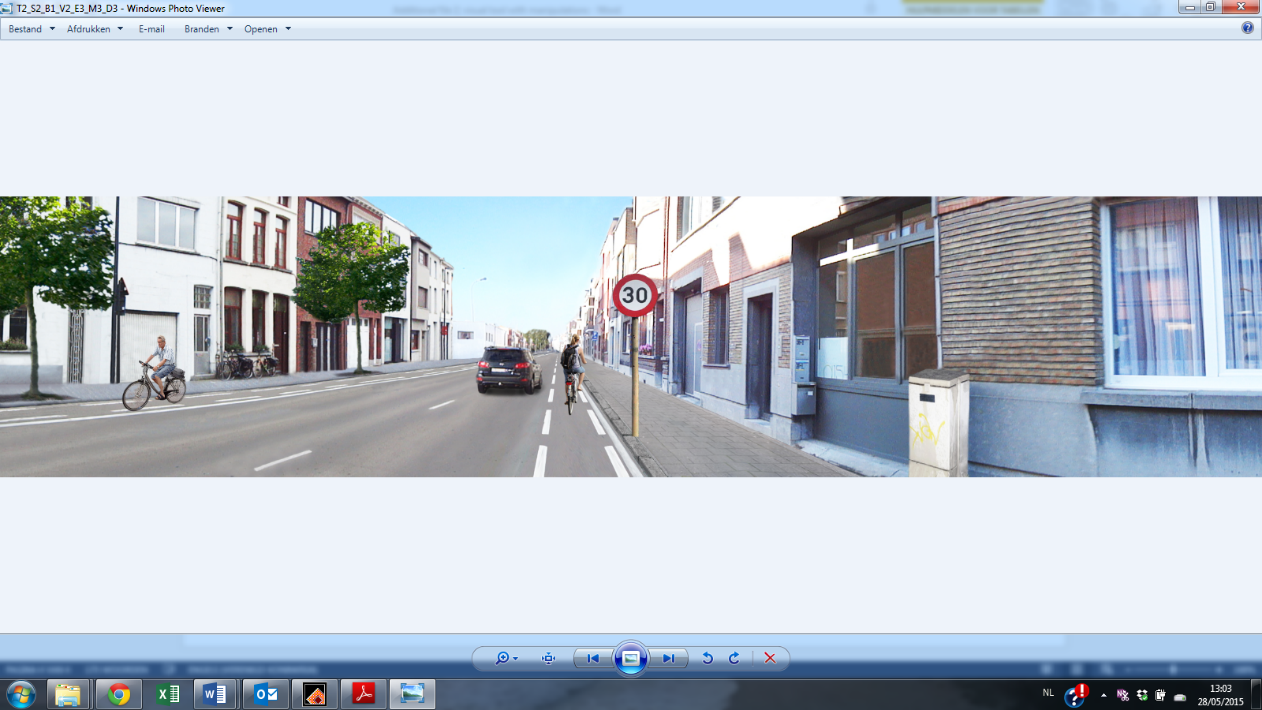 |
| Speed bump | 1. Absent 2. Present | 1. 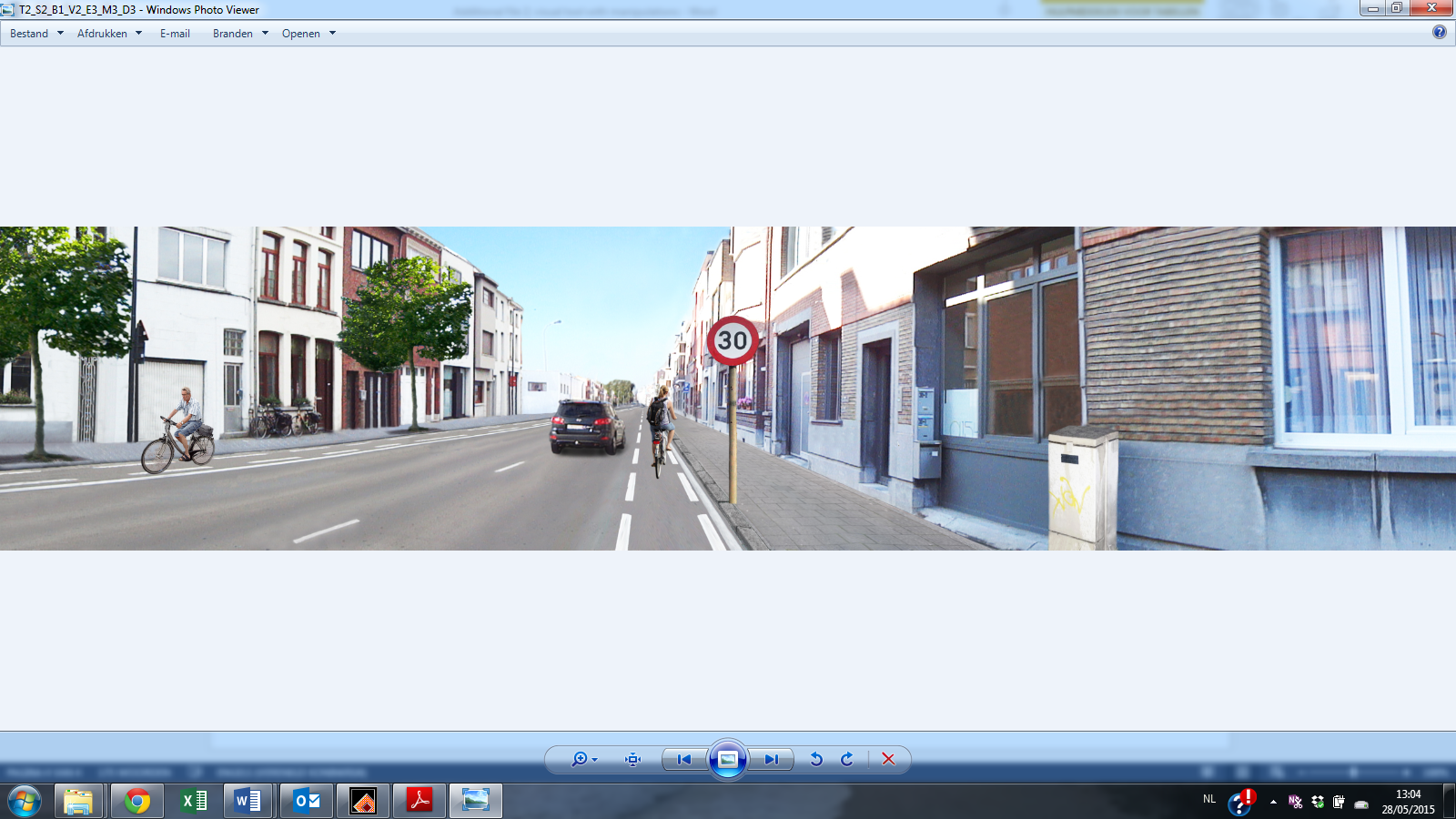   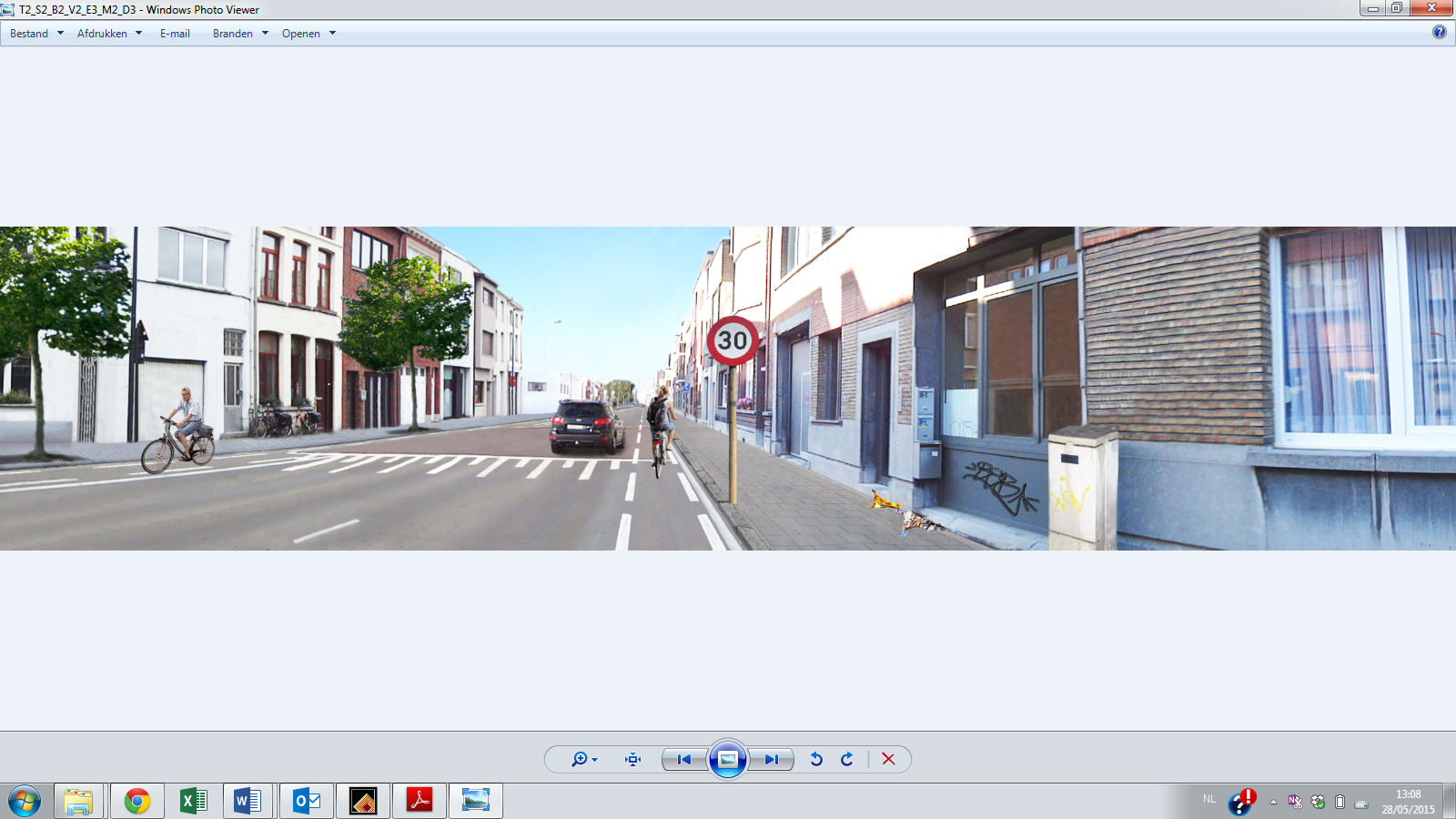 |
