# Supplementary material for: Creating Cycling-Friendly Environments for Children: Which Micro-Scale Factors Are Most Important? An Experimental Study Using Manipulated Photographs
Source: PLoS One. 2015 Dec 1;10(12):e0143302. doi: 10.1371/journal.pone.0143302 (PMC4666668; doi:10.1371/journal.pone.0143302)
Supplement: S2 Table — (DOCX) [file pone.0143302.s002.docx]

S2 Table : part-worth utilities within children’s subgroup 1

|  | **Part-worth utility** | **Standard Error** | **Lower 95% CI** | **Upper 95% CI** |
| --- | --- | --- | --- | --- |
| **Subgroup 1** |  |  |  |  |
| *Type 1* |  |  |  |  |
| Type 2 | 11.9 | 0.0 | 11.8 | 12.0 |
| Type 3 | 15.9 | 0.0 | 15.8 | 16.0 |
| Type 4 | 23.3 | 0.0 | 23.2 | 23.4 |
| Type 5 | 18.0 | 0.1 | 17.9 | 18.1 |
| Type 6 | 24.3 | 0.0 | 24.2 | 24.3 |
| *50 km/h* |  |  |  |  |
| 30 km/h | 2.8 | 0.0 | 2.7 | 2.9 |
| *absent* |  |  |  |  |
| present | 0.3 | 0.0 | 0.3 | 0.4 |
| *no trees* |  |  |  |  |
| two trees | -0.7 | 0.0 | -0.8 | -0.6 |
| four trees | 0.2 | 0.0 | 0.1 | 0.3 |
| *very uneven* |  |  |  |  |
| moderately uneven | 0.6 | 0.0 | 0.6 | 0.7 |
| even | 2.1 | 0.0 | 2.1 | 2.2 |
| *bad maintenance* |  |  |  |  |
| moderate maintenance | 2.3 | 0.0 | 2.2 | 2.4 |
| good maintenance | 2.3 | 0.1 | 2.2 | 2.4 |
| *4 cars + truck* |  |  |  |  |
| 3 cars | 0.9 | 0.0 | 0.8 | 1.0 |
| 1 car | 1.7 | 0.0 | 1.6 | 1.8 |
