# Supplementary material for: Creating Cycling-Friendly Environments for Children: Which Micro-Scale Factors Are Most Important? An Experimental Study Using Manipulated Photographs
Source: PLoS One. 2015 Dec 1;10(12):e0143302. doi: 10.1371/journal.pone.0143302 (PMC4666668; doi:10.1371/journal.pone.0143302)
Supplement: S4 Table — (DOCX) [file pone.0143302.s004.docx]

S4 Table : part-worth utilities within children’s subgroup 3

|  | **Part-worth utility** | **Standard Error** | **Lower 95% CI** | **Upper 95% CI** |
| --- | --- | --- | --- | --- |
| **Subgroup 3** |  |  |  |  |
| *Type 1* |  |  |  |  |
| Type 2 | 5.3 | 0.1 | 5.2 | 5.4 |
| Type 3 | 5.6 | 0.0 | 5.5 | 5.6 |
| Type 4 | 6.1 | 0.0 | 6.0 | 6.1 |
| Type 5 | 6.0 | 0.0 | 5.9 | 6.1 |
| Type 6 | 5.6 | 0.1 | 5.4 | 5.8 |
| *50 km/h* |  |  |  |  |
| 30 km/h | 1.1 | 0.0 | 1.0 | 1.2 |
| *absent* |  |  |  |  |
| present | 0.5 | 0.0 | 0.4 | 0.5 |
| *no trees* |  |  |  |  |
| two trees | 0.9 | 0.0 | 0.8 | 1.0 |
| four trees | 0.9 | 0.0 | 0.8 | 1.0 |
| *very uneven* |  |  |  |  |
| moderately uneven | 1.5 | 0.0 | 1.4 | 1.5 |
| even | 2.9 | 0.0 | 2.8 | 3.0 |
| *bad maintenance* |  |  |  |  |
| moderate maintenance | 6.5 | 0.0 | 6.4 | 6.5 |
| good maintenance | 9.7 | 0.1 | 9.5 | 9.8 |
| *4 cars + truck* |  |  |  |  |
| 3 cars | 1.9 | 0.0 | 1.8 | 2.0 |
| 1 car | 2.4 | 0.1 | 2.3 | 2.5 |
