# Supplementary material for: Creating Cycling-Friendly Environments for Children: Which Micro-Scale Factors Are Most Important? An Experimental Study Using Manipulated Photographs
Source: PLoS One. 2015 Dec 1;10(12):e0143302. doi: 10.1371/journal.pone.0143302 (PMC4666668; doi:10.1371/journal.pone.0143302)
Supplement: S5 Table — (DOCX) [file pone.0143302.s005.docx]

S5 Table : part-worth utilities within children’s subgroup 4

|  | **Part-worth utility** | **Standard Error** | **Lower 95% CI** | **Upper 95% CI** |
| --- | --- | --- | --- | --- |
| **Subgroup 4** |  |  |  |  |
| *Type 1* |  |  |  |  |
| Type 2 | 11.7 | 0.0 | 11.6 | 11.8 |
| Type 3 | 13.7 | 0.0 | 13.7 | 13.8 |
| Type 4 | 12.7 | 0.0 | 12.7 | 12.8 |
| Type 5 | 13.9 | 0.0 | 13.8 | 14.0 |
| Type 6 | 11.4 | 0.1 | 11.3 | 11.5 |
| *50 km/h* |  |  |  |  |
| 30 km/h | 1.5 | 0.0 | 1.4 | 1.6 |
| *absent* |  |  |  |  |
| present | -0.1 | 0.0 | -0.1 | 0.0 |
| *no trees* |  |  |  |  |
| two trees | 1.5 | 0.0 | 1.4 | 1.6 |
| four trees | 1.9 | 0.0 | 1.8 | 2.0 |
| *very uneven* |  |  |  |  |
| moderately uneven | 4.6 | 0.1 | 4.5 | 4.7 |
| even | 12.2 | 0.0 | 12.1 | 12.2 |
| *bad maintenance* |  |  |  |  |
| moderate maintenance | 4.4 | 0.0 | 4.4 | 4.5 |
| good maintenance | 4.7 | 0.0 | 4.7 | 4.8 |
| *4 cars + truck* |  |  |  |  |
| 3 cars | 3.1 | 0.0 | 3.0 | 3.2 |
| 1 car | 5.3 | 0.1 | 5.2 | 5.4 |
