# Supplementary material for: Creating Cycling-Friendly Environments for Children: Which Micro-Scale Factors Are Most Important? An Experimental Study Using Manipulated Photographs
Source: PLoS One. 2015 Dec 1;10(12):e0143302. doi: 10.1371/journal.pone.0143302 (PMC4666668; doi:10.1371/journal.pone.0143302)
Supplement: S7 Table — (DOCX) [file pone.0143302.s007.docx]

S7 Table : part-worth utilities within parents’ subgroup 1

|  | **Part-worth**  **utility** | **Standard Error** | **Lower 95%**  **CI** | **Upper 95%**  **CI** |
| --- | --- | --- | --- | --- |
| **Subgroup 1** |  |  |  |  |
| *Type 1* |  |  |  |  |
| Type 2 | 12.2 | 0.0 | 12.2 | 12.3 |
| Type 3 | 20.5 | 0.0 | 20.4 | 20.6 |
| Type 4 | 35.2 | 0.0 | 35.1 | 35.3 |
| Type 5 | 24.0 | 0.1 | 23.9 | 24.1 |
| Type 6 | 37.3 | 0.0 | 37.2 | 37.3 |
| *50 km/h* |  |  |  |  |
| 30 km/h | 4.0 | 0.0 | 4.0 | 4.1 |
| *absent* |  |  |  |  |
| present | 2.1 | 0.0 | 2.0 | 2.2 |
| *no trees* |  |  |  |  |
| two trees | 1.4 | 0.0 | 1.3 | 1.5 |
| four trees | 1.7 | 0.0 | 1.6 | 1.8 |
| *very uneven* |  |  |  |  |
| moderately uneven | 1.5 | 0.0 | 1.5 | 1.6 |
| even | 2.1 | 0.0 | 2.0 | 2.2 |
| *bad maintenance* |  |  |  |  |
| moderate maintenance | 1.6 | 0.0 | 1.5 | 1.6 |
| good maintenance | 2.8 | 0.0 | 2.7 | 2.8 |
| *4 cars + truck* |  |  |  |  |
| 3 cars | 1.1 | 0.0 | 1.0 | 1.1 |
| 1 car | 2.1 | 0.0 | 2.0 | 2.2 |
