# Supplementary material for: Creating Cycling-Friendly Environments for Children: Which Micro-Scale Factors Are Most Important? An Experimental Study Using Manipulated Photographs
Source: PLoS One. 2015 Dec 1;10(12):e0143302. doi: 10.1371/journal.pone.0143302 (PMC4666668; doi:10.1371/journal.pone.0143302)
Supplement: S9 Table — (DOCX) [file pone.0143302.s009.docx]

S9 Table : part-worth utilities within parents’ subgroup 3

|  | **Part-worth utility** | **Standard Error** | **Lower 95% CI** | **Upper 95% CI** |
| --- | --- | --- | --- | --- |
| **Subgroup 3** |  |  |  |  |
| *Type 1* |  |  |  |  |
| Type 2 | 15.1 | 0.1 | 15.0 | 15.3 |
| Type 3 | 24.2 | 0.1 | 24.1 | 24.3 |
| Type 4 | 28.0 | 0.1 | 27.9 | 28.2 |
| Type 5 | 23.4 | 0.1 | 23.3 | 23.5 |
| Type 6 | 30.0 | 0.2 | 29.6 | 30.3 |
| *50 km/h* |  |  |  |  |
| 30 km/h | 12.1 | 0.0 | 12.0 | 12.2 |
| *absent* |  |  |  |  |
| present | 2.7 | 0.1 | 2.6 | 2.8 |
| *no trees* |  |  |  |  |
| two trees | 0.2 | 0.0 | 0.1 | 0.2 |
| four trees | 0.5 | 0.0 | 0.4 | 0.6 |
| *very uneven* |  |  |  |  |
| moderately uneven | 1.0 | 0.0 | 0.9 | 1.1 |
| even | 1.3 | 0.0 | 1.2 | 1.4 |
| *bad maintenance* |  |  |  |  |
| moderate maintenance | 1.0 | 0.0 | 0.9 | 1.0 |
| good maintenance | 0.4 | 0.1 | 0.3 | 0.5 |
| *4 cars + truck* |  |  |  |  |
| 3 cars | -0.3 | 0.0 | -0.4 | -0.2 |
| 1 car | -0.4 | 0.0 | -0.5 | -0.4 |
