# Supplementary material for: Creating Cycling-Friendly Environments for Children: Which Micro-Scale Factors Are Most Important? An Experimental Study Using Manipulated Photographs
Source: PLoS One. 2015 Dec 1;10(12):e0143302. doi: 10.1371/journal.pone.0143302 (PMC4666668; doi:10.1371/journal.pone.0143302)
Supplement: S10 Table — (DOCX) [file pone.0143302.s010.docx]

S10 Table : part-worth utilities within parents’ subgroup 4

|  | **Part-worth utility** | **Standard Error** | **Lower 95% CI** | **Upper 95% CI** |
| --- | --- | --- | --- | --- |
| **Subgroup 4** |  |  |  |  |
| *Type 1* |  |  |  |  |
| Type 2 | 9.1 | 0.0 | 9.0 | 9.2 |
| Type 3 | 11.8 | 0.0 | 11.7 | 11.9 |
| Type 4 | 15.6 | 0.1 | 15.5 | 15.8 |
| Type 5 | 16.7 | 0.1 | 16.6 | 16.9 |
| Type 6 | 15.4 | 0.0 | 15.3 | 15.5 |
| *50 km/h* |  |  |  |  |
| 30 km/h | 3.6 | 0.1 | 3.5 | 3.7 |
| *absent* |  |  |  |  |
| present | 0.7 | 0.0 | 0.6 | 0.8 |
| *no trees* |  |  |  |  |
| two trees | 1.0 | 0.0 | 1.0 | 1.1 |
| four trees | 0.7 | 0.0 | 0.6 | 0.8 |
| *very uneven* |  |  |  |  |
| moderately uneven | 2.7 | 0.0 | 2.6 | 2.7 |
| even | 3.5 | 0.0 | 3.4 | 3.6 |
| *bad maintenance* |  |  |  |  |
| moderate maintenance | 0.6 | 0.0 | 0.5 | 0.7 |
| good maintenance | -0.8 | 0.1 | -1.0 | -0.7 |
| *4 cars + truck* |  |  |  |  |
| 3 cars | 7.3 | 0.1 | 7.2 | 7.4 |
| 1 car | 13.1 | 0.1 | 12.9 | 13.2 |
