# Supplementary material for: Creating Cycling-Friendly Environments for Children: Which Micro-Scale Factors Are Most Important? An Experimental Study Using Manipulated Photographs
Source: PLoS One. 2015 Dec 1;10(12):e0143302. doi: 10.1371/journal.pone.0143302 (PMC4666668; doi:10.1371/journal.pone.0143302)
Supplement: S11 Table — (DOCX) [file pone.0143302.s011.docx]

S11 Table : part-worth utilities within the total sample of parents

|  | **Part-worth utility** | **Standard Error** | **Lower 95% CI** | **Upper 95% CI** |
| --- | --- | --- | --- | --- |
| **Total Sample** |  |  |  |  |
| *Type 1* |  |  |  |  |
| Type 2 | 10.3 | 0.1 | 10.0 | 10.6 |
| Type 3 | 15.1 | 0.1 | 14.9 | 15.3 |
| Type 4 | 21.2 | 0.0 | 21.1 | 21.2 |
| Type 5 | 16.5 | 0.1 | 16.4 | 16.7 |
| Type 6 | 22.1 | 0.2 | 21.8 | 22.5 |
| *50 km/h* |  |  |  |  |
| 30 km/h | 3.1 | 0.0 | 3.0 | 3.2 |
| *absent* |  |  |  |  |
| present | 1.3 | 0.0 | 1.2 | 1.3 |
| *no trees* |  |  |  |  |
| two trees | 0.5 | 0.0 | 0.5 | 0.6 |
| four trees | 0.7 | 0.0 | 0.6 | 0.7 |
| *very uneven* |  |  |  |  |
| moderately uneven | 1.2 | 0.0 | 1.1 | 1.2 |
| even | 2.4 | 0.0 | 2.3 | 2.4 |
| *bad maintenance* |  |  |  |  |
| moderate maintenance | 2.0 | 0.0 | 1.9 | 2.0 |
| good maintenance | 2.8 | 0.1 | 2.7 | 3.0 |
| *4 cars + truck* |  |  |  |  |
| 3 cars | 1.4 | 0.0 | 1.3 | 1.4 |
| 1 car | 2.4 | 0.1 | 2.2 | 2.5 |
